# Histone H3.3 ensures cell proliferation and genomic stability during myeloid cell development

**DOI:** 10.1016/j.isci.2026.116501

**Published:** 2026-06-24

**Authors:** Sakshi Chauhan, Fuki Kudoh, Anup Dey, Keiko Ozato

**Affiliations:** 1 Division of Developmental Biology, National Institute of Child Health and Human Development, National Institutes of Health, Bethesda, MD 20892, USA; 2 Stem Cell Regulation Project, Tokyo Metropolitan Institute of Medical Science, 2-1-6 Kamikitazawa, Setagaya-ku, Tokyo 156-8506, Japan

**Keywords:** Molecular biology, Epigenetics, Immunology

## Abstract

Variant histone H3.3 is thought to be critical for the survival of many cells, since it is deposited in expressed genes. For example, *H3.3* deletion leads to embryonic lethality in mice. However, the requirement of H3.3 in the later stages of development has remained unclear. The aim of this work was to elucidate the role of H3.3 for the development of myeloid lineage, which is important for innate immunity. We conditionally knocked out (cKO) the H3.3 genes in myeloid progenitor cells differentiating into bone marrow-derived macrophages (BMDMs). Progenitor cells lacking H3.3 were defective in replication, suffered from extensive DNA damage, and underwent apoptosis. Surviving *H3.3*cKO cells expressed many interferon-stimulated genes throughout differentiation. Further, *H3.3*cKO BMDMs possessed chromatin accessible sites, histone posttranslational modifications consistent with the gene expression profiles, and retained general nucleosomal structure genome-wide. In summary, H3.3 is required for the proliferation of myeloid progenitor cells but is not totally indispensable for the differentiation of BMDMs.

## Highlights

• We utilized an *in vitro* model to differentiate bone marrow cells into macrophages
• *H3.3*cKO cells had extensive DNA damage and impaired cell proliferation
• *H3.3*cKO progenitors produced fewer macrophages that expressed inflammatory genes
• *H3.3*cKO cells retained the general nucleosomal structure genome-wide

## Graphical abstract

**Figure:**
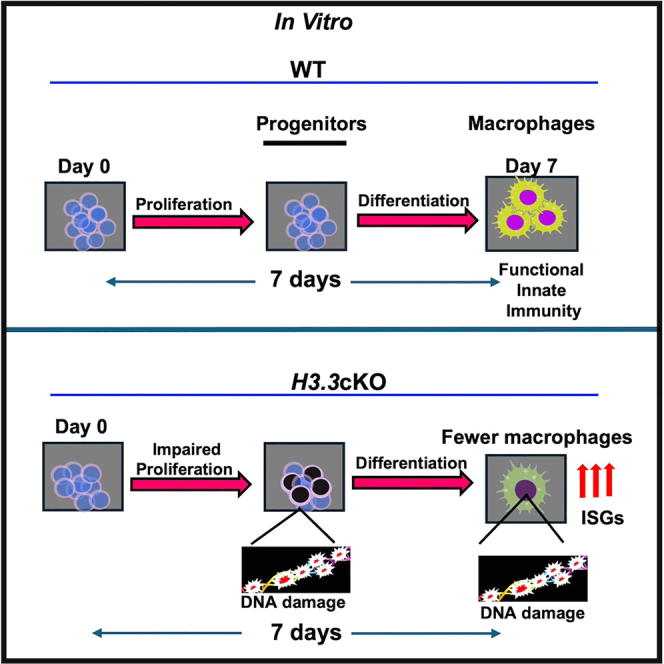

## Introduction

Histone H3.3, encoded by two genes, represents a variant deposited on nucleosomes of expressed genes throughout the cell cycle. This feature is distinct from that of core histones, H3.1 and H3.2, which are deposited during replication. Because of transcription-coupled deposition, H3.3 is implicated for epigenetic memory of expressed genes.^1^ In addition to actively expressed genes, H3.3 is found in the heterochromatin and telomeres.^2^^,^^3^

H3.3 is essential for embryonic development, since the deletion of H3.3 histone genes leads to early embryonic lethality, attributed to impaired proliferation.^4^

The requirement of H3.3 for later stages of development, however, appears more complex. In some models, the depletion of H3.3 histone genes does not totally abrogate developmental progression, while it alters the direction of differentiation. For example, *H3.3* deletion in hematopoietic stem cells is reported to result in myeloid lineage biased progenitor cells, displaying altered histone modification patterns.^5^ Deletion of *H3.3* in neuronal progenitor cells is shown to reduce the progenitor population but does not completely prevent the generation of postmitotic neurons.^6^ Recently it has been reported that H3.3 is downregulated during B lymphocytes development, and this process is necessary for plasma cells differentiation.^7^ Limited requirement of H3.3 for differentiation in some systems is surprising, however other non-replicative H3 variants may play partial redundancy with H3.3 in specific situations.

In this study, we investigated the role of H3.3 in myeloid cell development. The myeloid lineage originates from HSCs, which progress to myeloid progenitor cells, which then differentiate into terminally differentiated monocytes/macrophages. Myeloid lineage cells confer innate protection against pathogens and elicit inflammation, a role essential for life. We constructed mice in which both *H3f3a* and *H3f3b* genes were floxed, conditionally knocked out (cKO) in myeloid progenitors in bone marrow, using LysM-Cre, widely used to delete a gene of interest specifically in macrophages and granulocytes.^8^

We investigated how *H3.3* deletion affects development of bone marrow-derived macrophages (BMDMs) *in vitro*. We chose the *in vitro* model since it eliminates the potential effect of the incoming and outgoing cell population, which is unavoidable with *in vivo* models.

We show that *H3.3*cKO progenitor cells succumb to DNA damage and undergo apoptosis. Accordingly, fewer BMDMs were generated from *H3.3*cKO progenitors. We found that *H3.3*cKO BMDMs expressed many interferon-stimulated genes (ISGs), likely due to DNA damage. The ISG induction was mediated by the canonical interferon pathway, but did not depend on STING, IRF7, or MAVS(RIGI). Interestingly, *H3.3*cKO BMDMs possessed basic nucleosomal arrays, comparable to wild-type BMDMs. *H3.3*cKO BMDMs also displayed chromatin accessible sites and post-translational histone modifications concordant with the gene expression profiles. Together, H3.3 is obligatory for the proliferation of myeloid progenitor cells but not completely required for differentiation into postmitotic BMDMs.

## Results

### Cell cycle delay and apoptosis in *H3.3*cKO cells

Histone H3.3 is encoded by two separate genes, - *H3f3a* on chromosome 1 and *H3f3b* on chromosome 11 in the mouse. Each of these genes has 4 exons. To create *H3.3* ^*f*^^*l*^^*/f*^^*l*^ mouse, *LoxP* sites were inserted on flanking exons of *H3f3a* (exon 2) gene (Figure S1A) and *H3f3b* (exon 1–4) gene (Figure S1B) by homologous recombination. To create *LysM*^*cre/cre*^ conditional KO, *H3f3a*^*fl/fl*^*:H3f3b*^*fl/fl*^ mice were crossed with *LysM*^*cre/cre*^ mice. These mice were crossed with *LysM*^*cre/cre*^ mice to delete both *H3f3a* and *H3f3b* genes specifically in myeloid cells. *LysM*^*cre/cre*^ mice without floxed allele were shown to have normal myeloid lineage cells and produce BMDMs as those without *LysM*^*cre*^.^8^To further establish the validity of using *LysM*^*cre*^ mice, we confirmed that mice from *LysM*^*cre*^ without floxed allele produce BMDMs normally without DNA damage (Figure S1C).

At the genomic level, *H3f3a* and *H3f3b* deletion by *LysM*^*cre/cre*^ was confirmed by PCR screening (Figures S1D and S1E, and Table S1). The depletion of *H3.3* mRNA and protein in BMDMs was confirmed with the qRT-PCR, RNA seq (Figures S2A and B, and Table S2) and western blot analysis (Figure S1C), respectively. There were no phenotypic differences between WT(*H3f3a*^*fl/fl*^ *H3f3b*^*fl/fl*^) and *H3.3*cKO (*H3f3a*^*fl/fl*^ :*H3f3b*^*fl/fl*^*:LysM*^*cre/cre*^) mice.

**Figure 1:**
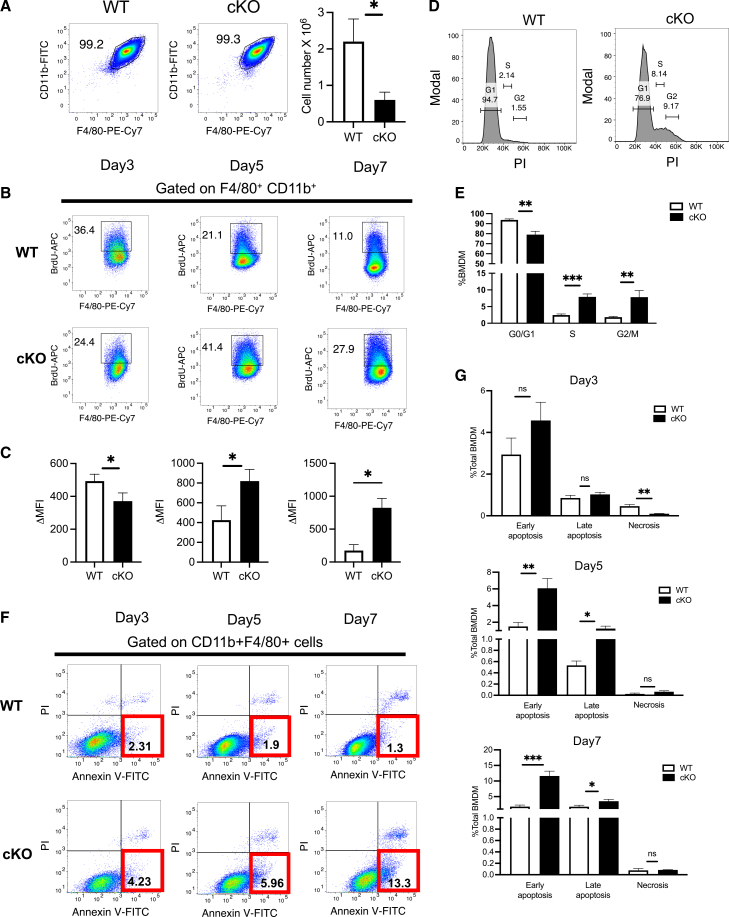
Proliferation defect and apoptosis in *H3.3*cKO BMDMs (A) Representative flow cytometry plots of bone marrow-derived macrophages upon staining with CD11b and F4/80 markers. Total number of BMDMs (right) obtained from WT and cKO BM cells. n (number of biological replicates) = 3, BMDMs were differentiated from 3 mice for each sample (WT and cKO). Unpaired *t* test was used to calculate *p* value (∗: *p* < 0.05). Bar graphs represent mean values ± SD. (B) Flow cytometry analysis of days 3, 5, and 7 WT and cKO cells upon incubation with BrdU. (C) Mean fluorescence intensities of BrdU were calculated for each day (3,5 and 7) in WT and cKO cells. n (number of biological replicates) = 3, (unpaired *t* test, ∗: *p* < 0.05). Bar graphs represent mean values ± SD. (D) Representative flow cytometric analysis of cell cycle in PI-stained WT and cKO BMDMs (day 7). (E) Percentage of WT and cKO cells was calculated for each phase of the cell cycle in PI-stained BMDMs. n (number of biological replicates) = 3, (Unpaired *t* test, ∗∗∗: *p* < 0.001 and ∗∗: *p* < 0.01). Bar graphs represent mean values ± SD. (F) Flow cytometry analysis of Annexin V-PI-stained WT and cKO cells to detect apoptosis. (G) Cell percentage was calculated for early apoptotic (Annexin V+, PI-), late apoptotic (Annexin V+, PI+ and necrotic (Annexin V-, PI+) WT, and cKO cells on days 3, 5, and 7 . n (number of biological replicates) = 3, unpaired *t* test was used to calculate *p* value (∗∗∗: *p* < 0.001; ∗∗: *p* < 0.01; and ∗: *p* < 0.05). Bar graphs represent mean values ± SD. ns - Not significant.

We performed flow cytometry analysis of BMDMs from WT and *H3.3*cKO mice using macrophage cell surface markers CD11b and F4/80. The number of *H3.3*cKO BMDMs was significantly lower, less than half of the WT counterpart (Figure 1A), which pointed to either a proliferation defect or increased apoptosis in *H3.3*cKO cells. Bone marrow cells, particularly progenitor cells, proliferate at early stages of culture and then differentiate into postmitotic BMDMs by day 7. As for peritoneal macrophages, we found no significant difference in their number between WT(*H3f3a*^*fl/fl*^ *H3f3b*^*fl/fl*^) and *H3.3*cKO(*H3f3a*^*fl/fl*^ *H3f3b*^*fl/fl*^*:LysM*^*cre/cre*^) (Figure S2D). This was expected as macrophages are continuously repopulated *in vivo*. CD11b and F4/80 expression profiles of WT and cKO cells on days 3, 5, and 7 are shown in Figure S3A. Both *H3.3* genes were deleted early on day 3 and 5 (Figures S3B and S3C). The deletion efficiency of *H3f3b* appeared variable, as we observed complete as well as the partial deletion of the gene. However, the observations were consistent among these cKO samples.

We examined the expression of histone *H3.1/2* genes in cKO cells to investigate if they compensate for H3.3 loss. Upon confirming that biological replicates of WT and cKO cells cluster together (Figure S3D), we found that the expression of core histone H3 genes appears somewhat higher in cKO cells on days 5 and 7, and lower on day 3 (Figure S3E). However, the differences were not statistically significant except for the lower expression of *Hist1h3c*, *Hist1h3g,* and *Hist2h3c2* genes in day 3 cKO cells (Figures S3F–S3H).

To investigate cell proliferation in *H3.3*cKO cells, we performed a BrdU incorporation assay for WT and *H3.3*cKO cells on day 3, day 5, and day 7 (schematic shown in Figure S3I). BrdU is incorporated into newly synthesized DNA by cells entering and progressing through the S phase of the cell cycle. Prolonged exposure of WT and cKO cells to BrdU led to the identification of actively cycling cell fractions. On day 3, BrdU uptake was significantly lower in *H3.3*cKO cells than in WT cells, indicating that *H3.3*cKO cells were deficient in timely DNA synthesis (Figures 1B and 1C). In line with reduced BrdU uptake, the expression of Ki67, a proliferation marker, was less in *H3.3*cKO cells than in WT cells on day 3 (Figures S4A–S4D). We observed, on the other hand, that *H3.3*cKO cells had higher BrdU uptake on days 5 and 7, suggesting that untimely replication persisted in some of the cKO cells (Figures 1B and 1C). Whereas Propidium iodide staining revealed that some of the *H3.3*cKO cells were in S phase, undergoing replication or at G2/M stage, in contrast to WT cells, which were mostly in G1 stage (Figures 1D and 1E). These results indicate that *H3.3*cKO cells were defective in cell cycle progression through S and G2/M stages, resulting in reduced production of total BMDMs.

It is possible that cells with defective proliferation may not survive and undergo apoptosis. To test this possibility, we performed Annexin V - PI staining. As shown in flow cytometry data in Figures 1F and 1G, *H3.3*cKO cells exhibited higher annexin V staining than WT cells. We also found that caspase 9, involved in apoptosis, was cleaved in *H3.3*cKO cells (Figure S4E).^9^ We also observed that poly (ADP-ribose) polymerase 1 (PARP-1) was cleaved in *H3.3*cKO cells, but not in WT cells (Figure S4E).^10^ These data led us to conclude that *H3.3*cKO cells are deficient in DNA replication, and a certain fraction of cells die due to apoptosis.

### *H3.3*cKO cells succumb to DNA damage

**Figure 2:**
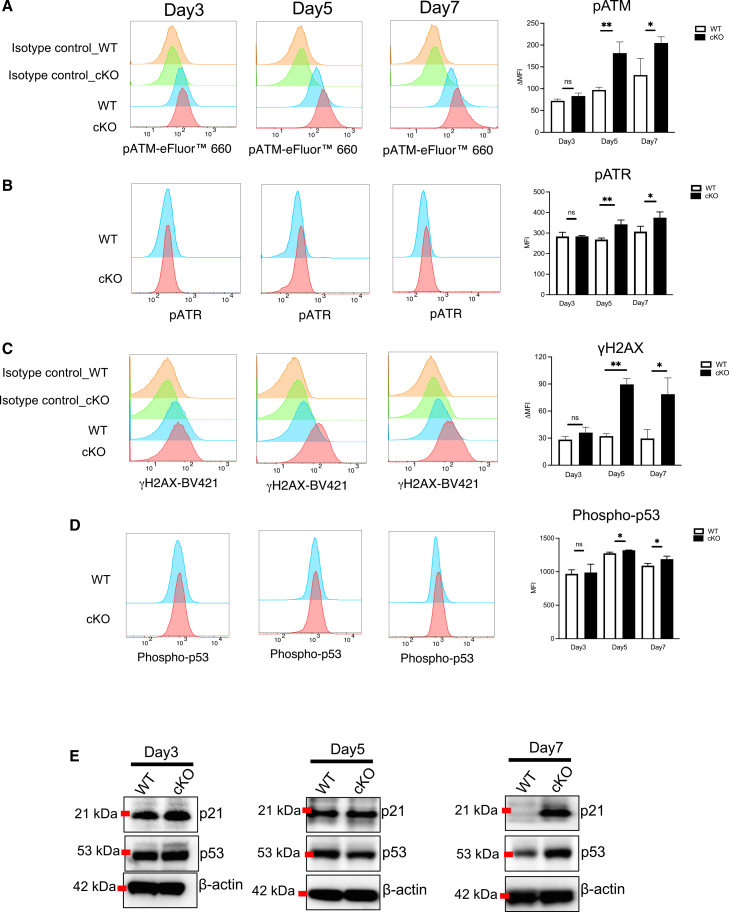
DNA damage in *H3.3*cKO BMDMs (A) Representative flow cytometric analysis of phosphorylated ATM on days 3, 5, and 7 in WT and cKO cells. Right side panel represents the average MFI values of pATM in three WT and three *H3.3*cKO cells ±SD. n (number of biological replicates) = 3. (Unpaired *t* test, ∗∗: *p* < 0.01 and ∗: *p* < 0.05, ns - Not significant). (B) Analysis of pATR levels via flow cytometry in WT and cKO cells on days 3, 5, and 7. Average MFI values of pATR in WT and cKO ±SD are shown on the right side. n (number of biological replicates) = 3. (Unpaired *t* test, ∗∗: *p* < 0.01 and ∗: *p* < 0.05, ns - Not significant). (C) Flow cytometric analysis of phosphorylated H2AX on days 3, 5, and 7 in WT and cKO cells. Quantification of MFI values in WT and cKO ±SD is presented in the bar graph on the right panel. n (number of biological replicates) = 3. (Unpaired *t* test, ∗∗: *p* < 0.01 and ∗: *p* < 0.05, ns - Not significant). (D) Time-course analysis of phosphorylated p53 (day 3, 5, and 7) in WT and cKO cells using flow cytometry. MFI values represent the average of three WT and three *H3.3*cKO cells ±SD. n (number of biological replicates) = 3. Unpaired *t* test was used to calculate *p* values (*p* < 0.01; ∗: *p* < 0.05, ns - Not significant). (E) Immunoblot detection of p53 and p21 protein in whole-cell extract of WT and cKO cells on day 3,5 and 7.

We surmised that the above-observed apoptosis is a consequence of DNA damage. Indeed, we found that ATM and ATR were phosphorylated in *H3.3*cKO cells (Figures 2A and 2B).

ATM and ATR are kinases, activated upon dsDNA breaks, which then initiate the DNA damage response (DDR) pathway.^11^^,^^12^ H2AX, a variant of histone H2A, is an immediate target of ATR and ATM. Phosphorylated H2AX, γH2AX is one of the most widely used markers for DNA damage.^13^ γH2AX is thought to bind to DNA damage sites, initiating DDR.^14^^,^^15^^,^^16^ p53 is a transcription factor that activates many downstream genes to regulate apoptosis and cell-cycle arrest, such as *p21*.^17^^,^^18^

Flow cytometry data in Figures 2A–2D demonstrated phosphorylation of ATM, ATR, H2AX, and p53 in *H3.3*cKO cells (quantification on the right). Western blot data in Figure 2E confirmed the overexpression of p21 in *H3.3*cKO cells on day 7, but not in WT cells. In line with this, we have previously shown that DNA damage occurs when cell cycle progression is inhibited.^19^ In sum, *H3.3*cKO cells sustain DNA damage and express multiple genes involved in DDR.

### Nucleotide synthesis genes are downregulated in *H3.3*cKO cells

**Figure 3:**
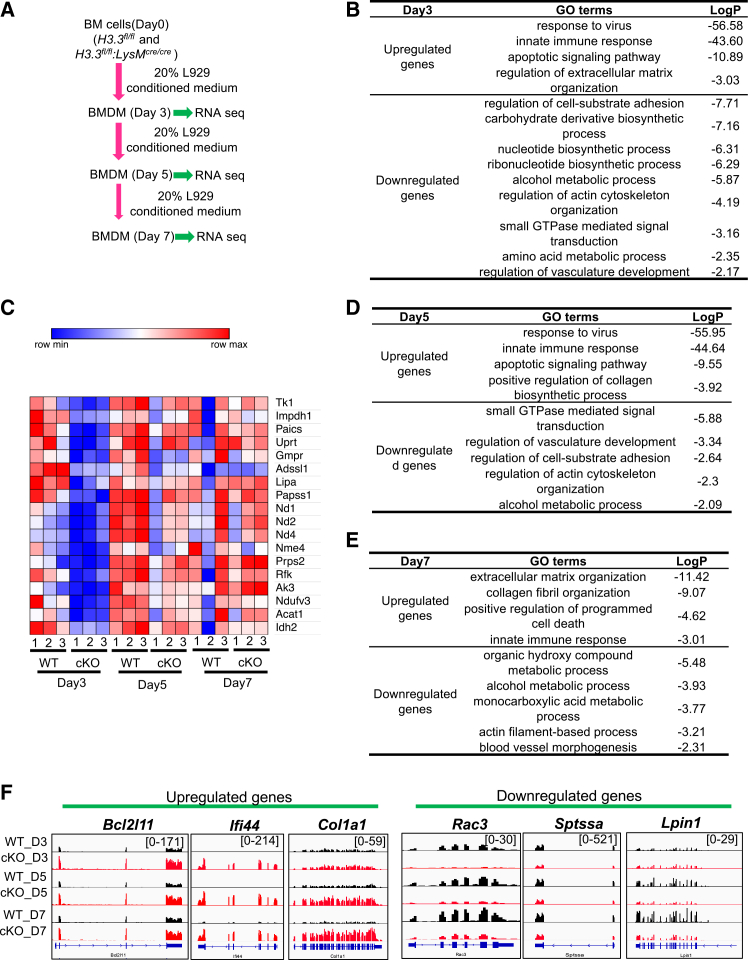
Transcriptome analysis of WT and cKO cells at different time points, i.e., days 3, 5, and 7 (A) Schematic of RNA seq with days 3, 5, and 7 BMDMs. (B) GO analysis of differentially expressed genes on day 3 cKO versus WT cells. (C) Heatmap shows RNA-seq normalized TPM values of nucleotide/ribonucleotide biosynthesis genes such as thymidine kinase 1(*Tk1*), adenylosuccinate synthase 1(*Adssl1*), inosine monophosphate dehydrogenase 1 (*Impdh1*), *Paics*, *Uprt* (uracil phosphoribosyl transferase), and *Gmpr* (guanosine monophosphate reductase), etc., on days 3, 5, and 7 cKO versus WT cells. (D) GO analysis of cKO vs. WT cells up and downregulated genes on day 5. (E) GO enrichment analysis of differentially expressed genes on day 7 cKO vs. WT cells. (F) RNA-seq IGV gene tracks of cKO upregulated genes (apoptotic gene *Bcl2l11*, ISG. *Ifi44* and collagen gene *Col1a1*) and cKO downregulated genes (small GTPase *Rac3*, sphingolipid biosynthesis gene *Sptssa,* and lipid synthesis gene *Lpin1*) show continuous up and downregulation, respectively, from day 3 cells to day 7 cells.

To delineate a mechanism of DNA damage in *H3.3*cKO cells, we undertook unbiased transcriptome profiling. RNA-seq was performed for *H3.3*cKO and WT cells on day 3, day 5, and day 7 (later on day 8) (Experimental Scheme in Figure 3A). On day 3, there were 1210 differentially expressed genes (upregulated in cKO - 781, downregulated in cKO - 429, FDR<0.05). GO analysis of the day3 DEGs revealed downregulation of genes important for nucleotide synthesis and ribonucleotide biosynthetic process (Figure 3B and Table S3) in cKO cells. Nucleotide metabolism is crucial for cell growth, as it supports DNA and RNA synthesis.^20^ Examples of genes in these categories include *Tk1*, *Impdh1*, *Adssl1*, *Uprt*, *Gmpr,* and *Paics,* which code for the enzymes involved in nucleotide synthesis (Figure 3C).^21^ Genes associated with carbohydrate biosynthesis, amino acid metabolism, and cytoskeletal organization were also downregulated (Figure 3B and Table S3). GO terms for upregulated genes included apoptosis-regulated genes (Figure 3B and Table S3). These data indicate that H3.3 is required for replication and cell cycle progression, and in its absence, apoptotic genes are activated. These results are consistent with the phenotypic data described above.

RNA-seq on day 5 and 7 BMDMs revealed 638 (upregulated in cKO - 498, downregulated in cKO - 140, FDR<0.05) and 627 (upregulated in cKO - 372, downregulated in cKO - 255, FDR<0.05) DEGs, respectively.

GO analysis of days 5 and 7 did not reveal nucleotide biosynthetic process and ribonucleotide biosynthetic process as the most prominent GO categories, suggesting that genes in these categories may be more sensitive to H3.3 deletion in early stages of bone marrow cell development (Figures 3D and 3E).

On the other hand, for upregulated genes, GO terms representing immune responses and response to viruses were found in *H3.3*cKO samples on days 3, 5, day 7 (Figures 3B–3E). IGV examples of upregulated (*Bcl2l11, Ifi44, Col1*) and downregulated (*Rac3, Sptssa,* and *Lpin1*) genes are presented in Figure 3F.

### Interferon-stimulated genes (ISGs) are upregulated in *H3.3*cKO BMDMs

**Figure 4:**
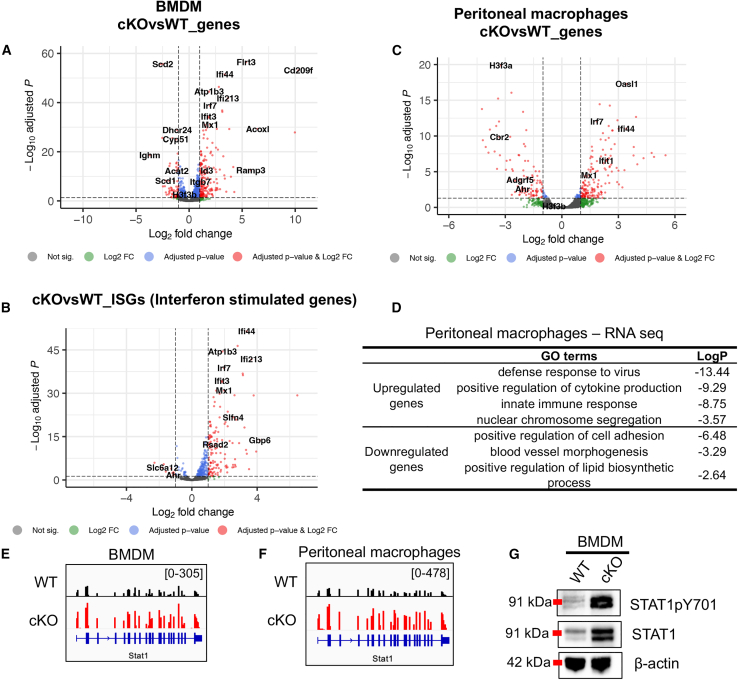
Transcriptome analysis of ISG expression in *H3.3*cKO BMDMs and peritoneal macrophages (A) Volcano plot for differentially expressed genes in cKO vs. WT bone marrow-derived macrophages (day 8 of cell differentiation). (B) Volcano plot for differentially expressed ISGs in cKO vs. WT BMDMs. (C) Volcano plot for differentially expressed genes in cKO vs. WT peritoneal macrophages. (D) GO analysis of differentially expressed genes in peritoneal macrophages upon *H3.3* deletion. (E) RNA seq IGV browser screenshot shows *Stat1* gene upregulation in cKO BMDMs. (F) Elevated RNA-seq read coverage over the *Stat1* gene in cKO peritoneal macrophages. (G) Western blot shows enhanced expression of STAT1 protein and its phosphorylated form in cKO BMDMs.

It was striking that genes representing innate immune responses and response to virus were upregulated in *H3.3*cKO cells at all stages of culture, from day 3 up to day 7. RNA-seq was also performed for BMDMs on day 8, which revealed 554 upregulated genes, while 309 genes were downregulated in *H3.3*cKO cells (FDR<0.05) (Figure 4A and Table S4). Out of 554 upregulated genes in cKO cells, many (311) were authentic ISGs, in that they were also induced after interferon-γ treatment. This is illustrated in volcano plots of Figures 4A and 4B (Table S5). The former depicts all DEGs and the latter ISGs only. Transcriptome analysis of *H3.3*cKO peritoneal macrophages also showed the upregulation of innate immune response genes, mostly ISGs (Figures 4C, 4D, and Table S4 and S5). Figures 4E–4G show enhanced mRNA and protein expression of *Stat1,* an ISG in *H3.3*cKO BMDMs and peritoneal macrophages, respectively. Extensive ISG expression would indicate that *H3.3*cKO BMDMs might be under an inflammatory state, given that many ISGs encode inflammation-inducing cytokines and chemokines.

### STING, MAVS, and IRF7 are dispensable for ISG induction in *H3.3*cKO BMDMs

We found ISG induction in *H3.3*cKO cells intriguing since DNA damage has been reported to cause ISG expression in various cells.^22^^,^^23^^,^^24^^,^^25^

In this scenario, small nucleotides produced by DNA damage would be sensed by pattern recognition receptors (PRRs), which initiate interferon expression. This would lead to full ISG expression by a second, feedback step through the engagement of type I interferon receptors (IFNAR).^26^^,^^27^

**Figure 5:**
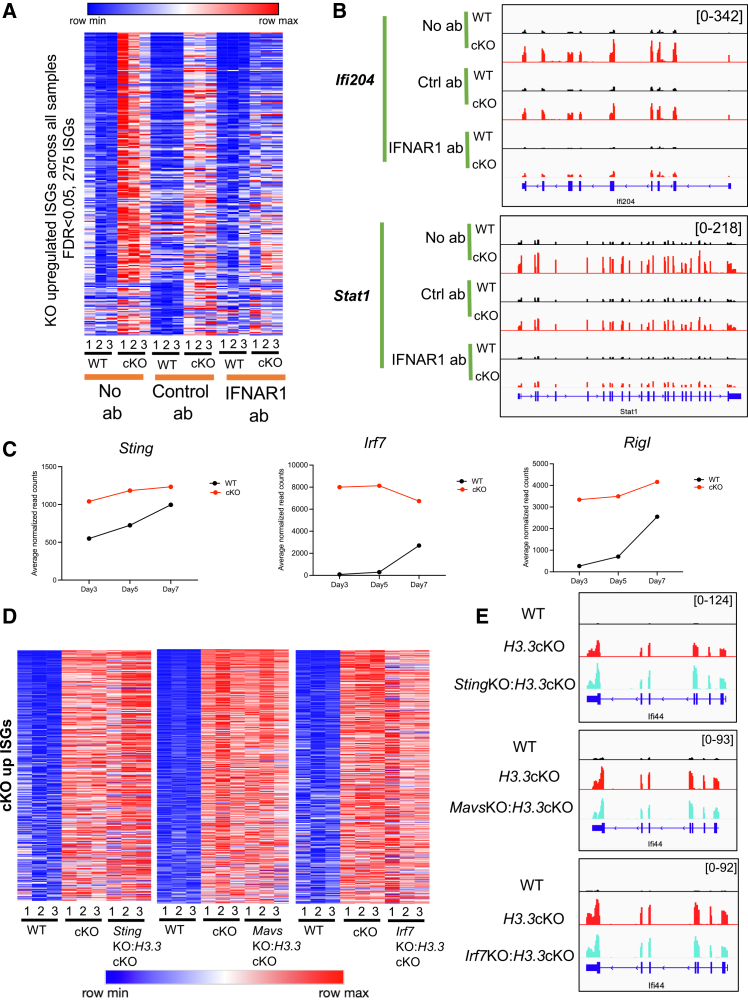
STING, IRF7, and MAVS are dispensable for antiviral response in cKO cells (A) Heatmap shows RNA-seq normalized read counts of cKO upregulated ISGs in WT and cKO BMDM samples for all three conditions (No antibody, control antibody, and IFNAR1 antibody). n (number of biological replicates) = 3. (B) RNA-seq gene tracks show lower expression of *Ifi204* and *Stat1* genes in cKO cells upon IFNAR neutralization in comparison to no antibody and control antibody. (C) RNA-seq normalized read counts of *Sting*, *Irf7,* and *RigI* genes in WT and cKO cells on days 3, 5, and 7. n (number of biological replicates) = 3. (D) Gene expression profiling heatmap shows the expression of cKO upregulated ISGs is unperturbed in *H3.3*cKO: *Sting* KO, *H3.3*cKO: *Irf7* KO, and *H3.3*cKO: *Mavs* KO BMDMs. (E) RNA-seq gene tracks show the expression of the *Ifi44* gene across WT, cKO, *H3.3*cKO: Sting KO, *H3.3*cKO: *Mavs* KO, and *H3.3*cKO: *Irf7* KO BMDMs.

To verify that ISG expression in *H3.3*cKO cells relies on canonical interferon induction pathways, we examined whether neutralization of IFNAR1 could inhibit ISG expression in *H3.3*cKO cells. Results in Figures 5A, 5B, S5A, and S5B confirmed that blocking of IFNAR receptor resulted in a marked reduction of ISG expression (Table S6). Thus, ISG expression in *H3.3*cKO cells occurs through a canonical interferon feedback pathway.

We then sought to find the mechanism that initiates the first interferon expression in *H3.3*cKO cells. Small nucleotides, endogenous or exogenous, are sensed by factors in the STING and RIGI-MAVS pathways.^28^^,^^29^^,^^30^ One of the pathways were of particular interest, since Li et al. recently reported that the cGAS-STING pathway is activated upon DNA damage, resulting in ISG induction^31^ Both *Sting* and *RigI*, themselves ISGs, were found upregulated in *H3.3*cKO cells (Figure 5C) To determine if one of these pathways plays a role, we constructed double knockout mice lacking STING and H3.3 (*H3.3*^*f*^^*l*^^*/*f^^l^:*LysM*^cre/cre^:*StingKO)* (verification of double KO Figures S5C–S5E) as well as those lacking MAVS and H3.3 (H3*.3*^*fL/*fL^:*LysM*^cre/cre^:*MavsKO* (verification of double KO in Figures S5F–S5H, and Table S7). RNA-seq was carried out for BMDMs generated from the double KO mice. Heat maps in Figure 5D (Table S8) showed that the deletion of *Sting* or *Mavs* genes did not abolish ISG expression in *H3.3*cKO cells. These results showed that STING and RIGI-MAVS pathways are not responsible for ISG induction in *H3.3*cKO cells. Finally, we tested if IRF7 plays a role in ISG expression in *H3.3*cKO cells. IRF7 is a transcription factor critical for host resistance as it activates many downstream ISGs [39]. IRF7 may also be activated by DNA damage.^32^^,^^33^ Double knockout mice lacking IRF7 and H3.3 (*H3.3*^*f*^^*l*^ ^/f^^l^:*LysM*^cre/cre^:*Irf7KO*) (verified in Figures S5I–S5K) were constructed, and RNA-seq was performed for BMDMs as above. Heat maps in Figure 5D (Table S8) again showed overall good ISG expression in double KO BMDMs, again indicating that IRF7 is not responsible for ISG expression in *H3.3*cKO BMDMs. MDS plots showed a concordance between *H3.3*cKO and double KOs (Figures S6A–S6C). We conclude that STING, MAVS, and IRF7 are not involved in ISG induction in *H3.3*cKO BMDMs. Together, factor(s) that initiates ISG induction in *H3.3*cKO cells remains to be identified (see discussion).

*H3.3* deletion has been reported to derepress endogenous retroviruses (ERVs) in mouse embryonic stem cells (ESCs) and hematopoietic stem cells.^5^^,^^34^ The role of H3.3 in repressing ERV, however, has been debated.^35^ Tal et al. reported that *H3f3a* and *H3f3b* differentially affect ERVs expression.^36^ Hoelper et al., showed that the H3.3 chaperon DAXX, but not ATRX, interacts with H3.3 not incorporated in chromatin to repress some ERVs in mouse ESCs.^37^ We examined the expression of ERVs in *H3.3cKO* BMDMs. As shown in the heatmap (Figures S6D–S6G), we observed a low-level increase in a few ERVs on day 3, but not on days 5 and 7, except ERVB2_1-I_MM-int, which was expressed at higher levels in *H3.3cKO* cells (77, 28, and 7-fold higher than WT on days 3, 5, and 7, respectively. Thus, H3.3 deletion does not seem to cause broad ERV reactivation in BMDMs under the conditions tested here.

### H3.3 deletion does not completely prevent the terminal differentiation of BMDMs

Bone marrow progenitor cells, upon several cycles of proliferation, differentiate into postmitotic, terminally differentiated BMDMs. It was somewhat surprising that *H3.3*cKO BMDMs exhibited typical macrophage morphology and adhered to the plate like WT BMDMs (Figure S7A). Moreover, they expressed many genes specifying macrophages, although they expressed ISGs in addition (Figures S7B–S7E). *H3.3*cKO BMDMs retained the ability to respond interferonγ and increased ISG expression (Figures S7F and S7G). Thus, the lack of H3.3 did not prevent bone marrow progenitor cells from developing into BMDMs, although it altered the transcriptome profiles to some degree. These results indicate that H3.3 is dispensable for many aspects of BMDM terminal differentiation. Similar observation was reported with respect to postmitotic neurons^6^; however, other cell types like ESCs require H3.3 for differentiation.^38^^,^^39^^,^^40^^,^^41^

### H3.3 localizes to genic regions

**Figure 6:**
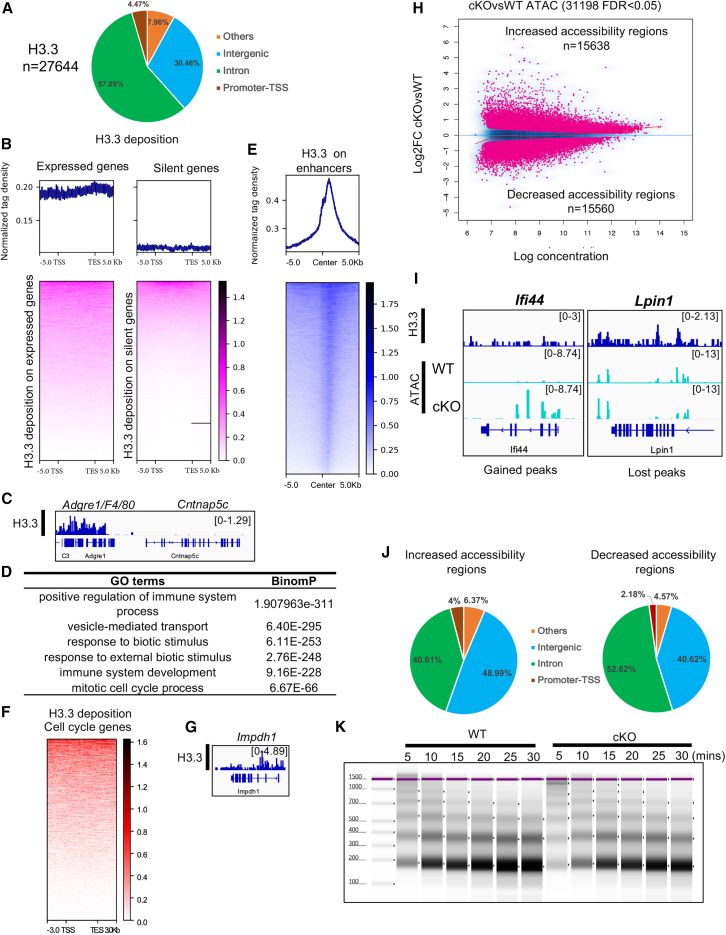
H3.3 distribution in *H3f3b*-HA BMDMs and chromatin accessibility analysis in cKO BMDMs (A) Pie chart shows H3.3 binding pattern in WT BMDMs (*H3f3b*-HA) upon CUT & RUN seq analysis. (B) Histogram and heatmap, derived from CUT & RUN seq, show binding of H3.3 on expressed genes and on silent genes/isoforms in *H3f3b*-HA BMDMs. (C) CUT & RUN seq IGV tracks show the deposition of H3.3 on expressed gene *Adgre1* and absence of H3.3 on silent gene *Cntnap5c*. (D) GO analysis of H3.3 binding peaks in *H3f3b*-HA BMDMs. (E) Profile plot and heatmap display the deposition of H3.3 on enhancer regions in *H3f3b*-HA BMDMs. (F) Heatmap shows H3.3 binding on cell cycle genes in *H3f3b*-HA BMDMs. (G) CUT & RUN seq gene tracks depict H3.3 binding on a cell cycle gene (*Impdh1*) in *H3f3b*-HA BMDMs. (H) MA plot depicts increased and decreased accessible regions (ATAC-seq) in cKO vs. WT BMDMs. (I) ATAC-seq gene tracks show increased accessible peaks at *Ifi44* and decreased accessible peaks at *Lpin1* gene in cKO cells. H3.3 binding at these genes is shown in the top panel. (J) Pie charts reveal the genome-wide distribution of cKO-gained and lost peaks upon chromatin accessibility analysis. (K) MNase-mediated digestion of chromatin from WT and cKO day 7 BMDMs.

To identify genome-wide H3.3 deposition sites, CUT & RUN seq was carried out using BMDMs (Day7) generated from knock-in mice in which the *H3f3b* gene was replaced by HA-tagged *H3f3b*.^42^ Mice carrying *H3f3b*-HA are fertile and have a normal immune system, making it as a convenient tool to determine genome-wide binding of H3.3 in cells *in vivo*.^42^ We found a total of 27645 H3.3-HA peaks, and genomic distribution puts the majority of the peaks on the gene bodies (57.09%) (Figure 6A). H3.3 deposition was higher on expressed genes than on silent genes (Figure 6B, 6C, and S8A), consistent with transcription-coupled incorporation. GO analysis revealed that H3.3 peaks are linked to immune regulation, representing the biological function of BMDMs (Figure 6D). Additionally, 30.46% of total H3.3 peaks were found in the intergenic regions, where H3.3 binding was enriched at presumed enhancer regions (Figure 6E). We also found some cell cycle genes occupied by H3.3, which may be attributed to the active transcription of cell cycle genes in the progenitor stage (Figure 6F, 6G, and S8B).

### Chromatin accessibility patterns match transcriptome profiles of *H3.3*cKO BMDMs

We performed ATAC-seq with day 7 BMDMs, which reveals genomic sites open to transcription factors. We found 31198 differential sites (FDR <0.05) between WT and *H3.3*cKO BMDMs, where *H3.3*cKO cells gained 15638 sites, while losing 15560 sites (Figure 6H). GO analysis revealed that the gained peaks were associated with innate immune response, cellular response to DNA damage stimulus, and apoptosis. Lost peaks were linked to the regulation of cell adhesion, regulation of leukocyte activation (Figures S8C and S8D). Many of the gained peaks were of the genes whose expression was gained in *H3.3*cKO, including ISGs. Examples of genes with gained or lost ATAC-seq peaks are presented in Figure 6I, namely *Ifi44* and *Lpin1*. As shown in Figure 6J, the majority of gained peaks were deposited in the intergenic regions, while lost peaks were mostly in the intronic region of *H3.3*cKO cells.

*De novo* motif analysis revealed enrichment of the Atf3(bZIP), Sfpi binding motifs in the gained peaks, while CEBPD and Sfpi1 binding motifs were also present in the lost peaks (Figure S8E). These results indicate that *H3.3*cKO cells were able to create new accessible sites, while eliminating others. These changes likely provided a basis for transcriptome programs characteristic of *H3.3*cKO cells.

Motif analysis in Figure S8E revealed Atf3 and CEBPD binding motifs associated with gained and lost peaks, respectively. This data is consistent with the results that *H3.3*cKO cells have DNA damage, become inflammatory, and likely lose macrophage functionality.^43^^,^^44^ Sfpi (PU.1) motifs were found in both gained and lost peaks. This is expected, since PU.1 is a master transcription factor for the development and function of macrophages.^45^

This observation prompted us to examine whether *H3.3*cKO cells maintain nucleosomal organizations without H3.3. We thus performed a micrococcal nuclease (Mnase) digestion assay with day 7 BMDMs. As shown in Figure 6K, the digestion patterns were very similar between WT and *H3.3*cKO samples at all time points tested. These data indicate that *H3.3*cKO cells were able to form nucleosomal organizations without H3.3.

### Genes upregulated in *H3.3*cKO cells gain H3K27ac and H3K36me3 marks

**Figure 7:**
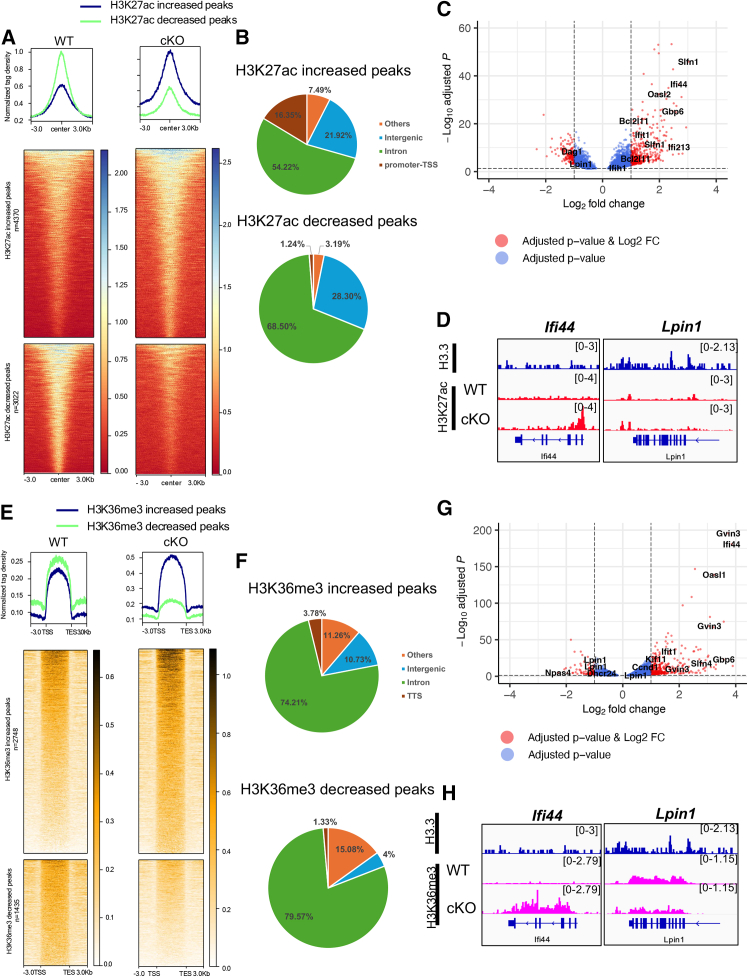
Differential distribution of H3K27ac and H3K36me3 in cKO cells (A) CUT & RUN seq derived histograms, and heatmaps of the H3K27ac densities for H3K27ac increased and decreased regions in WT and cKO cells. (B) Pie charts represent the genomic distribution of H3K27ac increased and decreased peaks. (C) Volcano plot depicts the correlation between changes in H3K27ac occupancy (based on CUT & RUN seq peaks) and corresponding changes in gene expression (based on RNA-seq data). The nearest genes for increased or decreased H3K27ac peaks correlate with genes up- or downregulated in *H3.3*cKO BMDM. (D) CUT & RUN seq IGV tracks of genes associated with H3K27ac increased (*Ifi44* gene), or H3K27ac decreased peaks (*Lpin1*) show H3K27ac distribution in WT and cKO cells. Top panel presents H3.3 binding on these genes in *H3f3b*-HA BMDMs. (E) CUT & RUN seq derived histograms, and heatmaps of the H3K36me3 densities for H3K36me3 increased and decreased regions in WT and cKO cells. (F) Genomic distribution of H3K36me3 increased and decreased peaks are shown in pie charts. (G) Volcano plot illustrates the correlation between changes in H3K36me3 occupancy (measured by CUT & RUN) and corresponding changes in mRNA levels (measured by RNA-seq). The nearest genes for increased or decreased H3K36me3 binding correlate with genes up- or downregulated in *H3.3*cKO BMDM. (H) CUT & RUN IGV tracks show gained and lost peaks of H3K36me3 in cKO BMDMs on *Ifi44* and *Lpin1* genes, respectively. Top panel shows H3.3 binding on these genes in *H3f3b*-HA BMDMs.

Post-translational histone modification marks (PTMs) that denote gene expression states are reported to be enriched on H3.3.^5^^,^^46^^,^^47^ We examined whether *H3.3* deletion results in changes in histone H3 modification marks, focusing on H3K27ac and H3K36me3. H3K27ac is a mark for active gene expression, enriched in enhancers of various sizes.^48^ CUT & RUN experiments were carried out to determine genome-wide H3K27ac sites in WT and *H3.3*cKO BMDMs. In WT cells, the majority of the H3K27ac peaks were found on gene bodies (Figures S9A and S9B). Differential binding analysis revealed that there were 4370 peaks with increased H3K27ac and 3022 peaks with reduced H3K27ac (FDR<0.05) in *H3.3*cKO cells (Figures 7A and S9C). A significant number of gained and lost peaks were found in the intron. Whereas 16.35% of the gained peaks were in the promoter-TSS region (Figure 7B). Volcano plot analysis revealed that the gained H3K27ac peaks were associated with genes involved in defense response to virus, programmed cell death, and DNA damage regulation. On the other hand, response to sterol and blood vessel morphogenesis was associated with lost peaks (Figure S9D). For instance, *Ifi44,* an ISG expressed in *H3.3*cKO cells, gained H3K27ac upon H3.3 deletion. Whereas *Lpin1* downregulated in *H3.3*cKO cells exhibited reduced H3K27ac peaks (Figure 7D).

The H3K36me3 mark has been linked with active transcription, DNA damage repair, and contribute to heterochromatin formation.^49^^,^^50^ CUT & RUN seq for H3K36me3 revealed the majority of the peaks on gene bodies in WT cells (Figures S9E and S9F). *H3.3*cKO cells had 2748 gained peaks and 1435 peaks of H3K36me3 for H3K26me3 (FDR<0.05) (Figures 7E and S9G). The majority of gained and lost peaks were in the gene bodies (Figure 7F). As expected, gained regions were found to be associated with response to interferon beta, chromosome organization, and mitotic cell cycle GO terms, while lost peaks were lipid biosynthetic process and cholesterol metabolic process genes (Figures 7G and S9H). An example in Figure 7H shows that *Ifi44* gained H3K36me3 marks in H3.3cKO cells, while *Lpin1* lost the mark, similar to H3K27ac above. Also, genes having H3.3 deposition possessed H3K27ac and H3K36me3 marks (Figures S9I and S9J). Moreover, genes upregulated in *H3.3*cKO cells gained H3K27ac as well as H3K36me3 marks, while those downregulated in *H3.3*cKO cells lost these modification marks. These results indicate that core histones H3.1 or H3.2, presumably substituting for H3.3, can gain PTMs associated with active gene expression.

## Discussion

The most salient findings we made in this study were (1) deletion of *H3.3* leads to DNA damage and apoptosis in bone marrow progenitors, yielding fewer terminally differentiated BMDMs, (2) *H3.3*cKO cells, both progenitor and BMDMs aberrantly expressed many ISGs, likely setting a prolonged inflammatory state, and (3) deletion of *H3.3*, however, did not prevent BMDMs from retaining genome-wide chromatin structure and post-translational histone modifications.

DNA damage in *H3.3*cKO progenitor cells was mediated by the canonical DDR response, starting with the activation of ATM and ATR, followed by the phosphorylation of H2AX (γH2AX), initiating assembly of DDR factors. In accordance, activated p53 was present in *H3.3*cKO cells, which led to the expression of many downstream DDR genes, including p21 (Figure S9K).^51^^,^^52^ In line with our findings, Jang et al., reported that the deletion of *H3.3* from early embryos causes DNA damage, preventing further development.^4^ It is possible that DNA damage ensues upon the deletion of *H3.3* in some cases. However, the role of H3.3 in preventing DNA damage has not been fully appreciated so far in other *H3.3* knockout models, including HSCs, where DNA damage was not shown.^5^^,^^6^^,^^53^

What is the mechanistic basis of DNA damage? Transcriptome data on day 3 showing that genes involved in nucleotide synthesis and ribonucleotide biosynthesis were downregulated in *H3.3*cKO cells strongly indicate that the deletion of *H3.3* interferes with DNA replication. *H3.3*cKO cells show reduced BrdU uptake on day 3. We found increased BrdU uptake in *H3.3*cKO cells on day 5 and day 7, which indicates that many of the *H3.3*cKO cells remain in S phase, unable to complete DNA replication, and are under replication stress. Given that replication stress is known to cause dsDNA breaks,^54^^,^^55^ it seems reasonable to assume that defective replication accounts for DNA damage in *H3.3*cKO cells.

We observed that many ISGs were upregulated in *H3.3*cKO cells throughout, from day 3 to day7/8, progenitor to differentiated BMDMs. This ISG induction was attributed to the authentic JAK/STAT pathway. Observed ISG induction may be a consequence of DNA damage, since ISG expression has been documented upon DNA damage caused by drugs and pathogen infection.^22^^,^^23^^,^^24^^,^^25^^,^^31^ To further understand ISG expression, we examined double KO mice lacking STING, MAVS, and IRF7, which recognize endogenous nucleotides and amplify ISG induction.^28^^,^^29^^,^^56^ We found none of these to be responsible for ISG induction in *H3.3*cKO cells. It is surprising that ISG induction was unaltered in the absence of STING, given that the cGAS-STING pathway is shown to play a critical role in ISG expression upon DNA damage.^31^ Therefore, an alternative mechanism exists that activates ISGs in *H3.3*cKO BMDMs. However, another possibility is that the absence of one pathway (for example, STING) creates a compensatory mechanism by the remaining factors (MAVS or IRF7), allowing for ISG expression, which cannot be ruled out. One possibility is DNA-PK. This kinase, activated by DNA damage, has been reported to stimulate ISG induction in a STING-independent manner.^57^^,^^58^ Given that DDR is activated in *H3.3*cKO cells, this possibility appears worth testing. Another possibility is a chromatin factor, Protein Arginine Methyltransferase 5 (PRMT5), which is reported to stimulate the transcription of inflammatory genes during replication stress by affecting ZNF326, a component of elongation.^59^ This mechanism may be relevant for *H3.3*cKO cells, given that they are defective in proliferation. Finally, ADA2, which catalyzes the deamination of adenosine, may play a role: ADA2 has been reported to repress inflammatory responses, and depletion of ADA2 activates inflammatory genes.^60^ It is possible that this system may be dysregulated in *H3.3*cKO BMDMs. It is important to determine the underlying mechanisms.

We also examined ERVs and found that only one ERV, ERVB2_1-I_MM-int, is highly upregulated in *H3.3*cKO BMDMs. While some other ERVs were somewhat higher than the basal level, it was less than 2-fold. At this point, the biological significance of ERV expression in BMDMs remains unclear. The role of H3.3 in ERV expression appears complex and may depend on cell types.^5^^,^^34^^,^^35^^,^^36^^,^^37^

It is interesting to note that a considerable number of *H3.3*cKO cells survived DNA damage and apoptosis and differentiated into BMDMs. *H3.3*cKO BMDMs displayed morphological traits such as WT BMDMs, and expressed many genes specific for macrophages, such as F4/80 and CD11b. These results indicate that H3.3 is not totally required for generating terminally differentiated BMDMs, although the deletion reduced the number of BMDMs, and changed gene expression, leading to extensive ISG expression. Supporting the view that H3.3 is partially, but not completely, required for terminal differentiation, Funk et al. reported that the deletion of *H3.3* from neuronal progenitor cells did not prevent the generation of postmitotic neurons, although it inhibited proliferation.^6^ In accordance with these findings, *H3.3*cKO BMDMs possessed nucleosomal arrays that were overall similar to those of WT cells. Consistent with a chromatinized genome, *H3.3*cKO BMDM had DNA accessible sites whose numbers were comparable to WT cells. Furthermore, nucleosomes of *H3.3*cKO BMDMs were decorated with post-translational histone modifications reflective of gene expression profiles. It is possible that in the absence of H3.3, core histones H3.1 and/or H3.2 compensate and are incorporated into expressed genes, allowing the maintenance of a chromatinized genome. It is also possible that another variant histone H3, yet to be identified, may be able to substitute for the lack of H3.3.^61^

In summary, this study highlights the critical requirement of H3.3 for DNA replication in the progenitor and development of the myeloid lineage.

### Limitations of the study

We observed DNA damage and impaired proliferation in the *H3.3* conditional KO progenitors and macrophages. However, the exact mechanism behind DNA damage in the absence of H3.3 is not very clear. Further studies about cell proliferation impairment in the absence of H3.3 are also required.

## Resource availability

### Lead contact

For further information and requests for resources and reagents, contact the lead contact, Keiko Ozato (ozatok@nih.gov).

### Materials availability

All materials and reagents generated in this study are available upon request.

### Data and code availability

• ATACseq data files: GEO: GSE285139.
• CUT & RUNseq data files: GEO: GSE285140.
• RNAseq data files: GEO: GSE285141.
• This paper does not report original code.
• Any additional information required to re-analyze the data reported in this paper is available from the lead contact upon request.

## Acknowledgments

We acknowledge the NICHD animal facility staff for caring for our mice and for other technical assistance. We gratefully acknowledge Kentaro Hoshi and other colleagues in our lab for technical advice and valuable discussions. This work was supported by the 10.13039/100000071NICHD Intramural programs ZIA HD008015-13.

## Author contributions

K.O. conceived the study and steered the project along with S.C.

S.C. performed flow cytometry, RNA-seq, CUT & RUN-seq, ATAC-seq, NGS data analysis, MNase digestion, and western blotting. S.C and F.K. performed qRTPCR and TEtranscripts data analysis. S.C. and A.D. analyzed flow cytometry data. A.D. and F.K. performed immunostaining and quantification of γH2AX. S.C. and K.O. prepared figures and wrote the manuscript.

## Declaration of interests

All authors declare no competing interests.

## STAR★Methods

### Key resources table

**Table:** 

REAGENT or RESOURCE	SOURCE	IDENTIFIER
**Antibodies**		

Mouse monoclonal H3.3	Sigma Aldrich	Cat # WH0003021M1; RRID:AB_1841959
Rabbit polyclonal anti-Histone H3	Abcam	Cat # ab1791; RRID:AB_302613
Rabbit monoclonal TFIIB	Abcam	Cat # ab109518; RRID:AB_10863223
Mouse monoclonal beta-Actin	Cell Signaling Technology	Cat # 3700; RRID:AB_2242334
Mouse monoclonal anti-Stat1 (N-terminus)	BD Biosciences	Cat # 611447; RRID:AB_2870038
Mouse monoclonal anti-Stat1(pY701)	BD Biosciences	Cat # 612232; RRID:AB_399555
Rabbit polyclonal anti-Parp1	Cell Signaling Technology	Cat # 9542; RRID:AB_2160739
Rabbit polyclonal anti-p53	Leica Biosystems	Cat # NCL-L-p53-CM5p; RRID:AB_2895247
Mouse monoclonal anti-CDKN1A	Santa Cruz Biotechnology	Cat # sc6246; RRID:AB_628073
Rabbit polyclonal anti-caspase-7	Cell Signaling Technology	Cat # 9492; RRID:AB_2228313
Mouse monoclonal anti-caspase-9	Cell Signaling Technology	Cat # 9508; RRID:AB_2068620
Rabbit polyclonal anti-caspase-3	Cell Signaling Technology	Cat # 9662; RRID:AB_331439
Rabbit polyclonal anti-HA tag	Abcam	Cat # ab9110; RRID:AB_307019
Rabbit polyclonal anti-Histone H3 (acetyl K27)	Abcam	Cat # ab4729; RRID:AB_2118291
Rabbit polyclonal anti-Histone H3 (tri methyl K36)	Abcam	Cat # ab9050; RRID:AB_306966
Rat monoclonal anti-CD16/CD32	Biolegend	Cat #101320; RRID:AB_1574975
Rat monoclonal Brilliant Violet 421™ anti- CD11b	Biolegend	Cat # 101235; RRID:AB_10897942
Rat monoclonal FITC anti- CD11b	Biolegend	Cat #101206; RRID:AB_312789
Rat monoclonal APC anti-F4/80	Biolegend	Cat # 123116; RRID:AB_893481
Rat monoclonal Brilliant Violet 421™anti-CD11b	BD Biosciences	Cat # 562605; RRID:AB_11152949
Rat monoclonal PE/Cyanine7 anti- F4/80	Biolegend	Cat # 123113; RRID:AB_893490
Rat monoclonal APC anti-Ki-67	Biolegend	Cat # 652405; RRID:AB_2561929
Mouse monoclonal BV421™ anti-H2AX (pS139)	BD Biosciences	Cat # 564720; RRID:AB_2738914
Mouse monoclonal BV421 IgG1, k Isotype Control	BD Biosciences	Cat # 562438; RRID:AB_11207319
Mouse Monoclonal eFluor™ 660 anti-phospho-ATM (Ser1981)	Thermo Fisher Scientific	Cat # 50-9046-42; RRID:AB_2574313
Rabbit polyclonal anti-ATR (phospho Thr1989)	GeneTex	Cat # GTX128145; RRID:AB_2687562
Rabbit polyclonal anti-phospho-p53 (Ser15)	Cell Signaling Technology	Cat # 9284; RRID:AB_331464
Goat anti-Rabbit IgG (H+L) Cross-Adsorbed Secondary Antibody, Alexa Fluor™ 633	Thermo Fisher Scientific	Cat # A-21070; RRID:AB_2535731
Rabbit monoclonal anti-phospho-Histone H2A.X (Ser139)	Cell Signaling Technology	Cat # 9718; RRID:AB_2118009
Goat anti-Rabbit IgG (H+L) Highly Cross-Adsorbed Secondary Antibody, Alexa Fluor™ 488	Thermo Fisher Scientific	Cat # A-11034; RRID:AB_2576217
Mouse monoclonal anti-IFNAR1(MAR1-5A3)	Thermo Fisher Scientific	Cat # 16-5945-85; RRID:AB_1210688
Mouse monoclonal IgG1 κ isotype control	Thermo Fisher Scientific	Cat # 14-4714-85; RRID:AB_470112

**Reagents**		

TheraPEAK ACK Lysing buffer (1X)	Lonza Bioscience	Cat # BP10-548E
DMEM and Ham’s F-12, 50/50 Mix	Corning	Cat # MT10092CV
FITC-Annexin V	BD Biosciences	Cat # 556420
Propidium Iodide Staining Solution	BD Biosciences	Cat # 556463
APC BrdU kit	BD Biosciences	Cat # 552598
Foxp3/Transcription Factor Staining kit	Thermo Fisher Scientific	Cat # 00-5523-00
Complete™, EDTA-free Protease Inhibitor Cocktail	Roche	Cat # 05056489001
Micrococcal Nuclease	NEB	Cat # M0247S

**Critical commercial assays**		

DNA clean & concentrator-5	Zymo Research	Cat # D4014
Quick-RNA Miniprep Kit	Zymo Research	Cat # R1055
QIAquick PCR Purification Kit	Qiagen	Cat # 28104
SuperSignal™ West Pico PLUS Chemiluminescent Substrate	Thermo Fisher Scientific	Cat # 34577

**Data and code availability**		

Raw data files ATAC-seq	This paper	GEO:GSE285139
Raw data files CUT&RUN-seq	This paper	GEO:GSE285140
Raw data files RNA-seq	This paper	GEO:GSE285141

**Experimental models - organisms/strains**		

Mouse: H3f3a^fl/fl^:H3f3b^fl/fl^: B6/J	This paper	N/A
Mouse: LysM^cre/cre^:B6.129P2-Lyz2^tm1(cre)Ifo^/J	The Jackson Laboratory	JAX: 004781
Mouse: H3f3a^fl/fl^:H3f3b^fl/fl^:LysM^cre/cre^: B6/J	This paper	N/A
Mouse: StingKO: B/6J × 129SvEv3	H. Ishikawa, & Barber G.N.^62^	https://pubmed.ncbi.nlm.nih.gov/18724357/
Mouse: MavsKO: B6;129-*Mavs*^*tm1Zjc*^/J	The Jackson Laboratory	JAX: 008634
Mouse: Irf7KO: B6.129P2-Irf7tm1Ttg	Honda K. et al.^56^	https://pubmed.ncbi.nlm.nih.gov/15800576/
Mouse: H3f3a^fl/fl^:H3f3b^fl/fl^:LysM ^cre/cre^:StingKO: B6/J	This paper	N/A
Mouse: H3f3a^fl/fl^:H3f3b^fl/fl^:LysM ^cre/cre^:MavsKO: B6/J	This paper	N/A
Mouse: H3f3a^fl/fl^:H3f3b^fl/fl^:LysM ^cre/cre^:Irf7KO: B6/J	This paper	N/A

**Oligonucleotides**		

See the Tables S1, S2, S3 and S4		

**Software and algorithms**		

GraphPad Prism	GraphPad Software	https://www.graphpad.com/features
Image J	Schneider, C.A.et al.^63^	https://imagej.net/ij/index.html
FlowJo	BD Biosciences	https://www.flowjo.com/
IGV	Broad Institute	https://igv.org/
Metascape	Zhou, Y. et al.^64^	https://metascape.org/gp/index.html#/main/step1
DiffBind	Ross-Innes, C.S. et al.^65^	https://hbctraining.github.io/Intro-to-ChIPseq/lessons/08_diffbind_differential_peaks.html
GREAT	McLean, C.Y. et al.^66^	http://great.stanford.edu/public/html/
Deeptools	Ramirez, F. et al.^67^	https://deeptools.readthedocs.io/en/latest/
TEtranscripts	Jin, Y. et al.^68^	https://www.mghlab.org/software/tetranscripts
Morpheus	Broad Institute	https://software.broadinstitute.org/morpheus/

### Experimental model and study participant details

#### Mice

All mice used in this study were bred and housed under specific pathogen-free conditions in a temperature and humidity-controlled environment with 12-hour light/dark cycle at the NICHD animal facility. The mice had *ad libitum* access to food and water. All animal protocols utilized in this study, were approved by the Animal Care and Use Committee at National Institute of Child Health and Human Development (Animal Study Program #17-044, #20-044 and #23-044). Mice used in this study were derived from a C57BL/6 genetic background. *H3.3*^*fl/fl*^ mice were crossed with *LysM*^*cre/cre*^ mice (strain # 004781, The Jackson Laboratory). *Sting* KO^62^ and *Irf7* KO^56^ mice were crossed with *H3.3*^*fl/fl*^*;LysM*^*cre/cre*^ mice to generate *StingKO:H3.3cKO* and *Irf7KO:H3.3cKO* mice respectively. *Mavs* KO mice (Strain # 008634, The Jackson Laboratory) were backcrossed to *H3.3*^*fl/fl*^*;LysM*^*cre/cre*^ (C57BL/6 background) strain over at least six generations before use in experiments. Deletion of *Sting*, *Mavs* and *Irf7* genes was confirmed using PCR, qRTPCR and RNA sequencing. HA tagged *H3f3b* mice^42^ were used for H3.3 CUT & RUN experiment. We used 8 to10-weeks old male and female mice in this study.

#### Bone marrow derived macrophages culture

Bone marrow cells were collected by flushing the femurs and tibias of 8 to 10-week-old C57BL/6 mice and passed through filter. Collected cells were treated with ACK lysis buffer and subsequently washed with PBS. Cells were cultured in the DMEM/F-12 media supplemented with 10% FBS, 1X antibiotic and 20% L929 conditioned media for 6-8 days.

### Method details

#### Flow cytometry analysis of cell surface receptors

Single cell suspensions were made from peritoneal macrophages and bone marrow derived macrophages. After blocking with CD16/CD32 antibody, cells were stained with respective antibodies at 4°C for 30 min. Subsequently, cells were washed multiple times with the washing buffer (5 % FBS in 1X PBS), resuspended in the buffer and observed in BD LSRFortessa™. The analysis was done by using FlowJo software.

#### Flow cytometry - intracellular staining

For Ki67 and γH2A.X staining, cell surface markers-stained cells were resuspended in BD cytofix/cytoperm buffer and incubated at 4°C for 30 minutes. Subsequently cells were washed with 1X BD perm/wash buffer. Washed cells were resuspended in antibody containing 1X BD perm/wash buffer and incubated for 20 mins at room temperature. Fixed and permeabilized cells were further washed and resuspended in perm/wash buffer and staining buffer respectively. Stained cells were analyzed by LSR Fortessa (BD Biosciences).

For phosphorylated ATM, ATR and p53, eBioscience™ Foxp3 / Transcription Factor Staining kit was used as per manufacture’s protocol.

#### Annexin V - PI assay

Cells were harvested and washed with cold PBS. Further cells were resuspended in binding buffer (0.01M HEPES pH-7.4, 0.14M NaCl, 2.5 mM CaCl_2_) at a concentration of 10^6^ cells per ml. Cells were further aliquoted as 100 μl (10^5^ cells) per tube. 5μl of FITC Annexin V and 2.5 μl of PI (1mg/ml) were added in the tubes. After gentle vortexing, cells were incubated at room temperature for 15 min in dark. Cells were further diluted in the binding buffer and flow cytometry analysis was performed using LSR Fortessa (BD Biosciences). Data were analyzed using FlowJo software.

#### BrdU based cell proliferation assay

For identification of actively proliferating cells, cells were exposed to BrdU for longer hours. Cells were incubated with 50 μg/ml BrdU for 15 hrs. and 24 hrs. for earlier time points (day3 and day5) and day7 respectively. After incubation, cells were washed and stained with cell surface markers. Stained cells were fixed and permeabilized using BD Pharmingen™ APC BrdU Kit. Subsequently cells were stained with BrdU antibody and analyzed by flow cytometry.

#### Propidium iodide (PI) staining

Cells were fixed with 70% ethanol for two hours at 4°C and subsequently washed with PBS. RNase A treatment was given at 37°C for 30 mins. After washing the fixed cells with PBS, cells were resuspended in PI containing PBS and incubated for 30 min at room temperature. Cells were analyzed by flow cytometry.

#### Immunocytochemistry- γH2AX staining

Day 7 cells/BMDMs were fixed, permeabilized and incubated with Phospho-Histone H2A.X (Ser139) (Rabbit mAb) for 2 hours at room temperature. Anti-rabbit FITC was used for detection. Images were captured using a Zeiss LSM 880 confocal microscope. Mean fluorescence intensity of γH2AX signal was analyzed using ImageJ/Fiji.^63^

#### Quantitative real time PCR

Total RNA was isolated using Trizol. cDNA was synthesized using High-capacity cDNA reverse transcription kit (Applied Biosystems, Thermo Fisher Scientific). Quantitative real time PCR was performed using Fast SYBR™ Green Master Mix (Applied Biosystems, Thermo Fisher Scientific). Quantitative differences were calculated using the 2ˆ-(ΔΔCt) method relative to *Gtf2b*.

#### Blocking of IFNAR1 receptor

WT and cKO BM cells were incubated with F12/DMEM supplemented with 20% L929 conditioned medium. IFNAR1 monoclonal antibody or IgG1 κ isotype control antibodies were added at a concentration of 10 μg/mL at 37 °C. Antibodies were added since day0 and media was supplemented with fresh antibodies every 12 hrs. On Day 6, the media containing antibodies were removed, and cells were lysed with trizol.

#### RNA seq and data analysis

For BMDM, total RNA was prepared using Quick-RNA Mini prep Kit (Zymo Research). RNA integrity was confirmed by running the RNA sample on an Agilent Bioanalyzer RNA 6000 Nano chip to determine the RNA integrity number. mRNA enrichment was done using NEBNext® rRNA depletion kit. RNA seq libraries were prepared using NEBNext® Ultra™ II RNA library prep kit.

Peritoneal macrophages were isolated using miltney kit. Total RNA was purified by trizol extraction. Library was prepared using SMART-Seq v4 Ultra Low Input RNA Kit (Takara bio USA, CA) with 7-cycle amplification.

Samples were pooled and sequenced (paired end) on the Illumina NextSeq 500 and NextSeq2000.

For data analysis, quantification, and quality-control pipeline (CCBR pipeliner v4.0.6) was used. Briefly, adapter sequences are removed using Cutadapt and paired-end reads were aligned to the *Mus musculus* reference genome mm10 using STAR aligner. Gene and isoform expression levels were quantified using RSEM. For differential expression analysis SARTools1.8.1^69^ were used with q value of <0.05. Heatmaps were depicted with Morpheus (https://software.broadinstitute.org/morpheus). For transposable element analysis, we used TE transcripts package.^68^ Metascape was used for GO analysis.^64^

Genes with baseMean >20 (RNA seq) were considered expressed genes (n=12922). Remaining genes and isoforms were considered as silent genes. Cell cycle gene list was used as described.^19^ To define ISGs, we stimulated WT BMDMs with interferon γ for different time points (2,4,6,8,10 and 12 hrs.) and performed RNA seq. All the upregulated genes (merged and duplicates were removed) were defined as ISGs.

All known mouse genes were downloaded from UCSC table browser.

#### CUT & RUN seq and data analysis

CUT & RUN was performed as reported earlier.^70^ Cells were collected and washed twice with the buffer (20 mM HEPES pH 7.5, 150 mM NaCl, 0.5 mM Spermidine, 1x Protease inhibitor cocktail). Concanavalin A-coated magnetic beads were washed twice with the binding buffer (20 mM HEPES-KOH pH-7.9, 10 mM KCl, 1 mM CaCl_2_,1 mM MnCl_2_). Cells were resuspended in wash buffer, mixed with the bead suspension. The cell-bead mix was rotated for 10 min at room temperature, placed on magnetic rack and liquid was discarded. Beads were further dislodged in antibody buffer (wash buffer containing 2mM EDTA, 0.02% digitonin and respective antibody). Antibody incubation was done overnight at 4°C. After incubation, beads were placed on magnetic rack and all the liquid was removed. Subsequently, beads were washed with digitonin buffer (wash buffer containing 0.02 % digitonin). Further, beads were resuspended in pA-MNase (700ng/ml) containing digitonin buffer and incubated on tube rotator for 1 hour at 4 C. After 1 hour, beads were washed twice with digitonin buffer, resuspended in 100 μl of digitonin buffer, and placed on a cold block to reach 0°C. 3 μl of 100 mM CaCl_2_ was mixed in the bead suspension which was further incubated at 4°C for 30’. The reaction was stopped by adding 100 μl of 2X stop buffer (0.34 M NaCl, 20 mM EDTA, 4 mM EGTA, 0.02 % digitonin, 50 μg/ml RNase A, 50 μg/ml glycogen, 20 pg/ml yeast spike in DNA). To release the fragments, beads were incubated at 37 C for 10′, centrifuged at 4°C for 5’. The supernatant was collected from the tubes on the magnetic rack. For histone antibodies, DNA was extracted by using Qiagen spin column, while in the case of transcription factors, PCI based extraction was done after adding 0.1% SDS and 150 μg/ml of proteinase K in the sample and incubating it at 70 C for 10‘. (Spike in).

CUT & RUN seq libraries were prepared using NEBNext® Ultra™ II DNA library kit (Illumina). Samples were pooled and paired end sequencing (25 bp) was performed on the Illumina NextSeq 500.

For data analysis, CUT & RUN -seq reads were mapped to the mouse reference genome (mm10) using Bowtie2 v.2.3.4.1 with the following parameters: --local --very-sensitive-local --no-unal --no-mixed --no-discordant --phred33 -I 10 -X 700. The peak calling was performed using homer. The bam files were converted to normalized bigWig files by using scale factor, deeptools bamCoverage command.^67^ DiffBind was used to analyze differential binding.^65^ We used GREAT to further identify the genes associated with the peaks.^66^

#### ATAC seq and data analysis

ATAC seq was performed as described previously.^71^^,^^72^ Briefly, 50,000 cells were lysed to prepare crude nuclei extract. The nuclei were resuspended in transposition reaction mix and incubated at 37^0^ C for 30’. Transposed DNA was collected using DNA Clean & Concentrator™-5 purification columns. Eluted DNA was amplified using NEBNext® Ultra™ II Q5 Mater Mix and size selected (Negative and positive selection). Purified DNA samples were assessed using Bioanalyzer High sensitivity DNA analysis kit. Samples were subsequently pooled and sequenced using paired-end sequencing on the Illumina NextSeq 500. Paired reads were aligned to mouse reference genome mm10 using Bowtie2-2.5.1 Peak calling was performed by using Genrich with default settings. To identify differential ATAC-seq peaks between WT and cKO samples, R package DiffBind was used. MA plot, cluster correlation analysis plots were generated using DiffBind package.^65^ For motif analysis, homer was used.

#### MNase digestion

Day7 BMDMs were washed with ice cold PBS and resuspended in buffer (50mM NaCl, 10mM Tris-Cl, pH-7.5, 5 mM MgCl_2_, 1mM CaCl_2_, 0.2 % NP-40, 1X protease inhibitor cocktail). After the lysis, nuclei were resuspended in buffer and incubated with 1U MNase for indicated time points (5, 10, 15, 20, 25 and 30 mins). The enzymatic reaction was stopped by addition of EGTA. After phenol, chloroform, isoamyl alcohol treatment of the reaction, aqueous phase DNA was collected and further purified by Qiagen purification kit.

#### Acidic extraction of histones

Acidic extraction of histones was performed as reported (https://www.abcam.com/protocols/histone-extraction-protocol-for-western-blot). Briefly, WT and KO cells were washed with ice cold PBS. Subsequently cells were lysed in triton extraction buffer (PBS containing 0.5 % Triton X 100 (v/v), 2 mM phenylmethylsulphonyl fluoride (PMSF), 0.02 % (w/v) NaN_3_) for 10 min on ice. Nuclei pellets were collected by centrifugation at 4°C for 10 min at 6500 g. Nuclei were further washed in triton extraction buffer. Washed nuclei were resuspended in of 0.2N HCl and acid extracted overnight at 4°C. Histones (supernatant) were collected by centrifugation at 4°C for 10 min at 6500 g. Acid extracted histones were further neutralized with 2M NaOH.

#### Whole cell extract and nuclear extract preparation

For cell lysates preparation, cells were lysed in Radio Immunoprecipitation Assay (RIPA) buffer (25 mM Tris-HCl, pH7.6, 150 mM NaCl (sodium chloride), 1% NP-40, 0.1% SDS (sodium dodecyl sulfate), 0.1% sodium deoxycholate) with protease and phosphatase inhibitors for 1h at 4°C. Whole cell lysates were clarified by centrifugation at 12,000 rpm for 10min at 4°C. Nuclear extracts were prepared as described.^73^

#### Western blotting

Cell lysates and histones were separated on NuPAGE^TM^4-12 % and 12% Bis-Tris gel respectively. Proteins were subsequently transferred to PVDF membrane (Millipore). Blocking was performed at room temperature for 1 hour with 2.5% BSA. Blots were incubated overnight with primary antibody at 4°C and then washed twice for 10 min each. Secondary antibody incubation was performed at room temperature for 1 hour. To remove nonspecific binding, blots were washed again and then incubated with ECL for 5 min and analyzed with Azure c600 Biosystems.

### Quantification and statistical analysis

All statistical analyses were performed using GraphPad Prism software. Data are presented as mean ± SD from independent experiments performed in triplicate (biological replicates) except Figure S5B where error bars represent standard error of mean. Statistical significance was defined as *p* < 0.05. Significance was calculated using unpaired *t* test except Figure S5B where *p* values were calculated using ordinary one-way ANOVA test. Specific statistical tests, and *p* values for each experiment are provided in the corresponding figure legends.

## Supplemental information

Document S1. Figures S1–S10, and Tables S1, S2, and S7

Table S3. Differentially expressed genes at different time points i.e., Day3, 5 and 7 in cKO vs. WT cells, related to Figure 3

Table S4. Differentially expressed genes in day8 BMDMS and peritoneal macrophages (FDR<0.05), related to Figure 4

Table S5. List of ISGs, related to Figure 4

Table S6. Differentially expressed genes upon IFNAR1 blocking in cKO vs. WT cells, related to Figure 5A

Table S8. List of differentially expressed genes in StingTKO, MavsTKO and Irf7TKO BMDMs, related to Figure 5D
