# Supplementary material for: Histone H3.3 ensures cell proliferation and genomic stability during myeloid cell development
Source: iScience. 2026 Jun 24;29(7):116501. doi: 10.1016/j.isci.2026.116501 (PMC13320441; doi:10.1016/j.isci.2026.116501)
Supplement: Document S1. Figures S1–S10, and Tables S1, S2, and S7 [file mmc1.pdf]

iScience, Volume *29*

Supplemental information

Histone H3.3 ensures cell proliferation and genomic stability during myeloid cell development

Sakshi Chauhan, Fuki Kudoh, Anup Dey, and Keiko Ozato

Figure S1

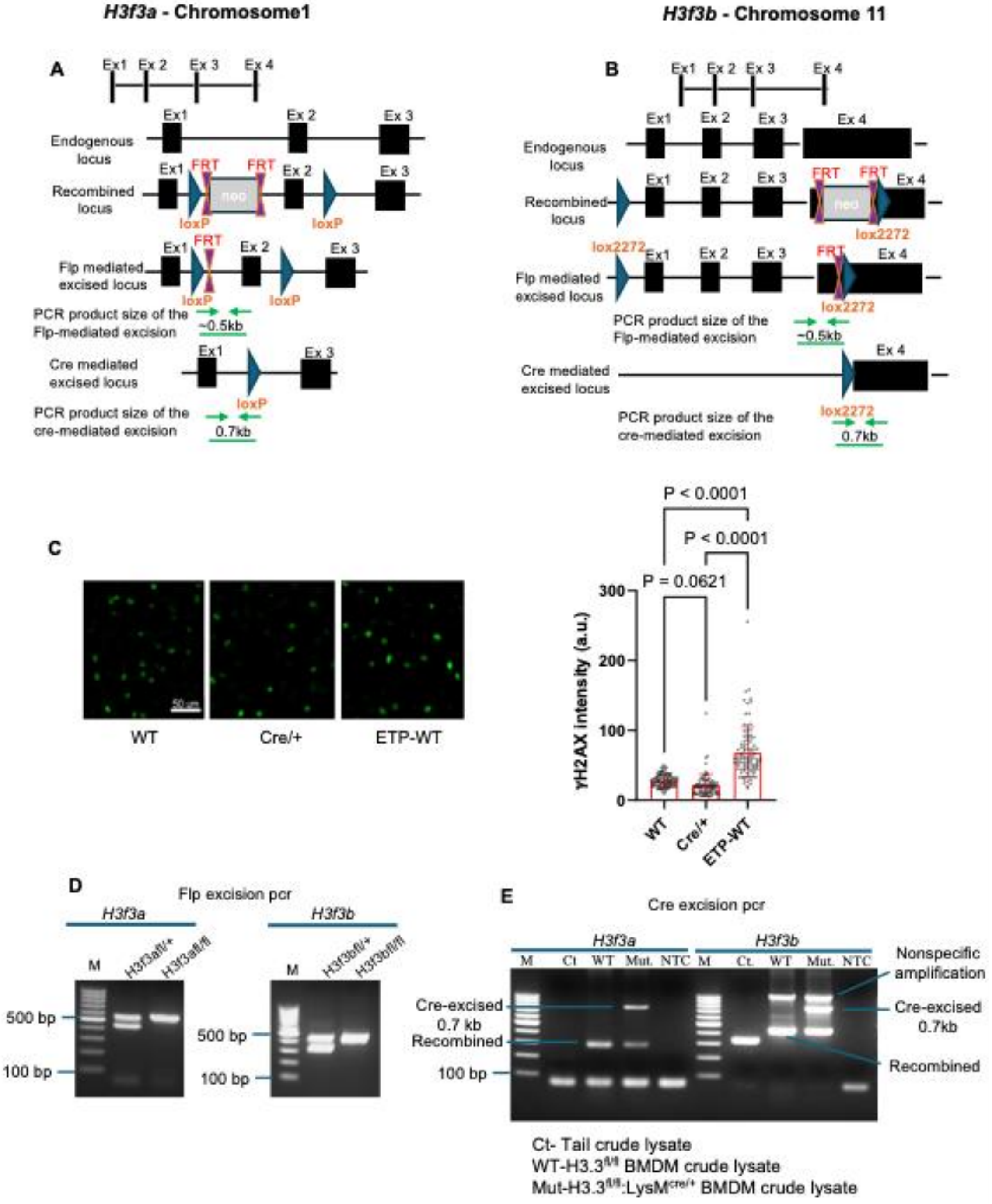

H3f3a - Chromosome1
H3f3b - Chromosome 11
A
B
Endogenous locus
Recombined locus
Flp mediated excised locus
PCR product size of the Flp-mediated excision
~0.5kb
Cre mediated excised locus
PCR product size of the cre-mediated excision
0.7kb
loxP
FRT
lox2272
neo
C
WT
Cre/+
ETP-WT
P < 0.0001
P < 0.0001
P = 0.0621
γH2AX intensity (a.u.)
D
Flp excision pcr
E
Cre excision pcr
Cre-excised 0.7 kb
Recombined
Nonspecific amplification
500 bp
100 bp
Ct- Tail crude lysate
WT-H3.3fl/fl BMDM crude lysate
Mut-H3.3fl/fl:LysMcre/+ BMDM crude lysate

Figure S1. Recombination and LysMcre based conditional deletion of histone *H3.3* genes, Related to Figure1

A. Schematic representation of *H3f3a* and

B. *H3f3*b alleles, created for this study. Green arrows represent primer positions.

C. Confocal microscopy images (scale bar - 50μm) representing γH2A.X staining in BMDMs from WT ($H3f3a^{fl/+}$:$H3f3b^{fl/fl}$: $LysM^{+/+}$), $LysM^{cre/+}$($H3f3a^{fl/+}$:$H3f3b^{fl/+}$:$LysM^{cre/+}$) and ETP-WT ($H3f3a^{fl/+}$:$H3f3b^{fl/fl}$:$LysM^{+/+}$) samples. Etoposide (ETP) (10μM for 24 hours) is positive control. Right panel depicts quantification of γH2A.X signal. n (number of biological replicates) =3, Unpaired t test was used to calculate p values.

D. Flp mediated excised products (~0.5kb) were amplified upon PCR with heterozygous and homozygous floxed H3.3 genes (*H3f3a* and *H3f3b*).

E. LysMcre excised (0.7kb) band amplification upon cre excision PCR with control, WT($H3f3a^{fl/fl}$:$H3f3b^{fl/fl}$) and mutant ($H3f3a^{fl/fl}$:$H3f3b^{fl/fl}$:$LysM^{cre/+}$) BMDMs confirmed the deletion of *H3f3a* and *H3f3b* genes in Mut/cKO BMDMs.

Figure S2

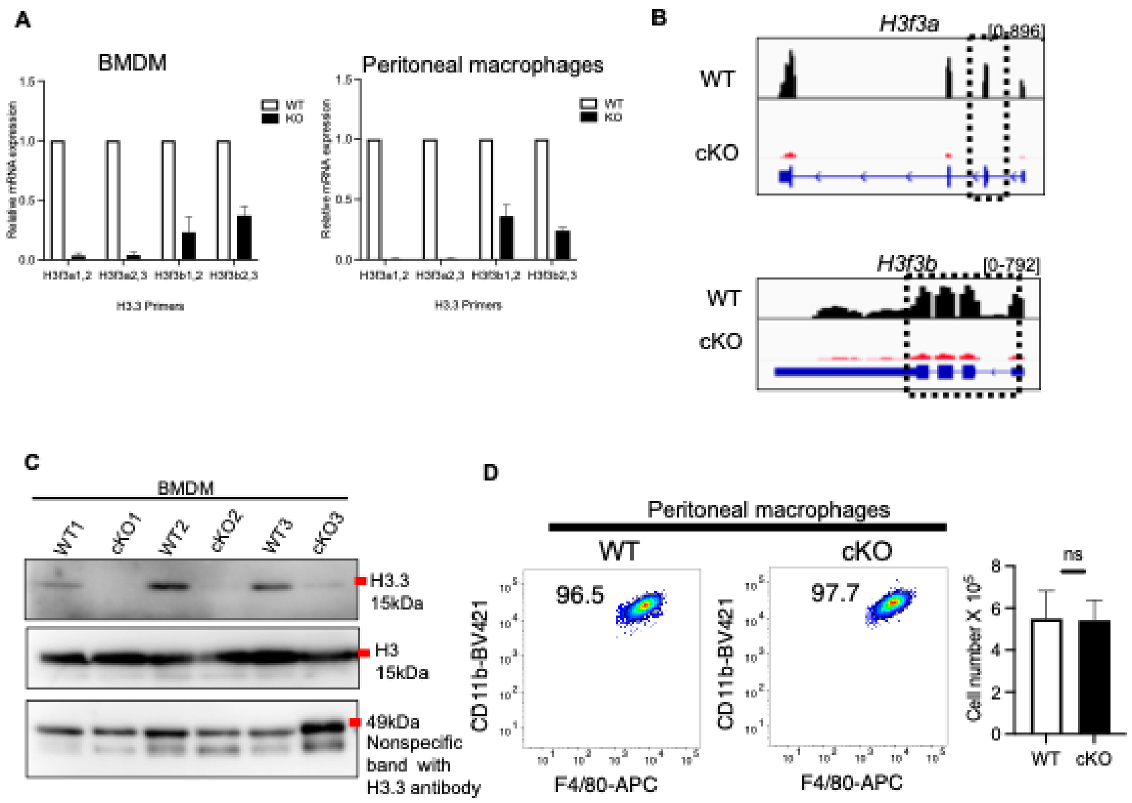
A
BMDM
Peritoneal macrophages
WT
KO
Relative mRNA expression
H3f3a1,2
H3f3a2,3
H3f3b1,2
H3f3b2,3
H3.3 Primers
B
H3f3a
[0-896]
WT
cKO
H3f3b
[0-792]
C
BMDM
WT1
cKO1
WT2
cKO2
WT3
cKO3
H3.3
15kDa
H3
15kDa
49kDa
Nonspecific band with H3.3 antibody
D
Peritoneal macrophages
WT
cKO
96.5
97.7
CD11b-BV421
F4/80-APC
ns
Cell number X 10⁵
WT
cKO

Figure S2. Confirmation of histone H3.3 deletion and flow cytometric analysis of WT and H3.3cKO peritoneal macrophages, Related to Figure1

A. qRTPCR confirmation of *H3.3* deletion at RNA level in day 7 cKO BMDMs and cKO peritoneal macrophages. n (number of biological replicates) =3
B. RNA-seq gene tracks show *H3f3a* and *H3f3b* gene deletion in cKO cells. Dotted lines represent deleted genomic regions in cKO cells.
C. Western blot confirmation of *H3.3* deletion in day 7 cKO BMDMs at protein level.
D. Representative flow cytometry plots for peritoneal macrophages upon staining with CD11b and F4/80 markers. Total number of peritoneal macrophages (right) were obtained from WT and cKO mice, n (number of biological replicates) =3. Unpaired t test was used to calculate p values. (ns – Not significant).

Figure S3

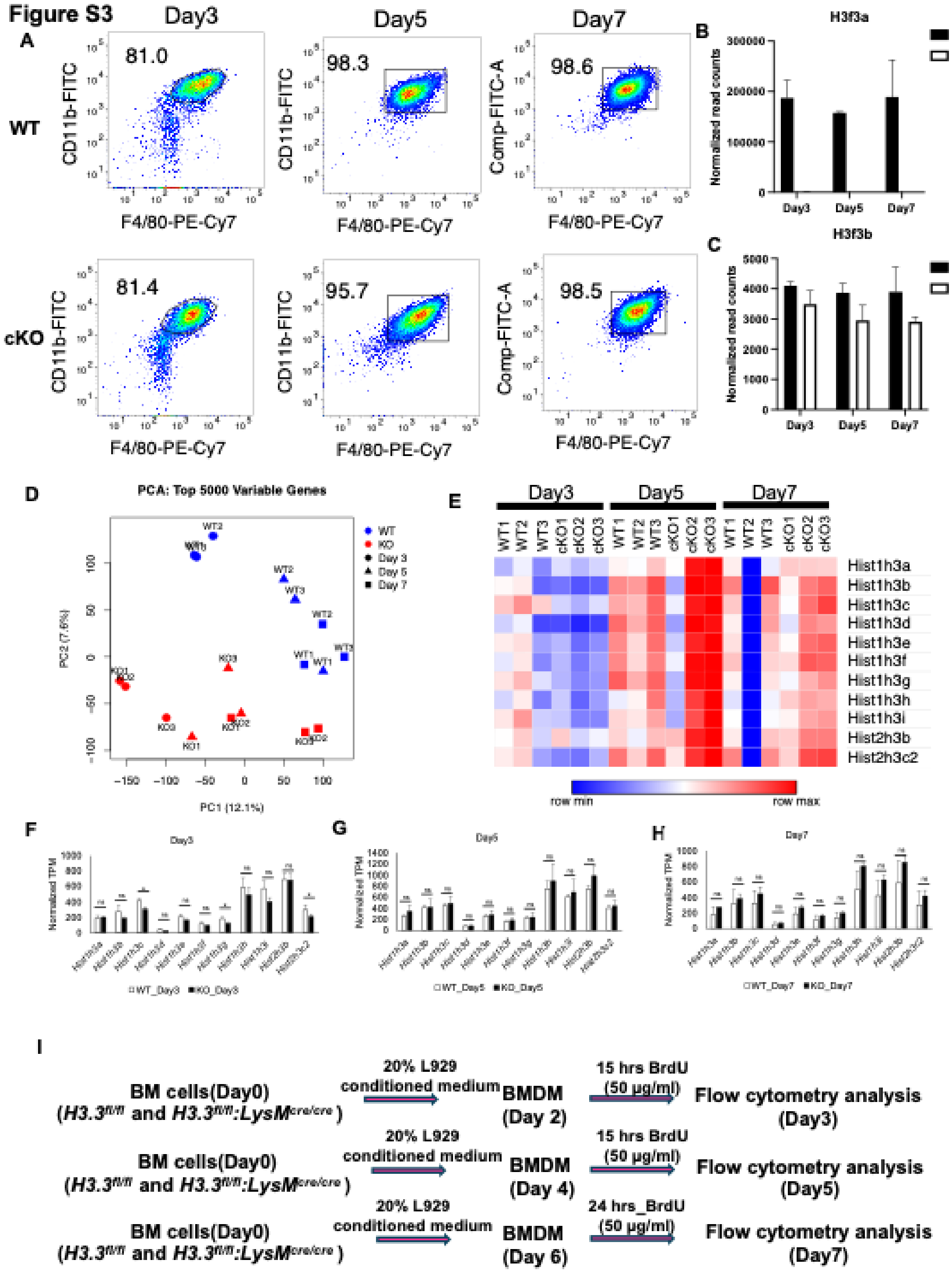
A
Day3
Day5
Day7
WT
cKO
CD11b-FITC
Comp-FITC-A
F4/80-PE-Cy7
81.0
98.3
98.6
81.4
95.7
98.5
B
H3f3a
Normalized read counts
WT
KO
Day3
Day5
Day7
C
H3f3b
D
PCA: Top 5000 Variable Genes
PC1 (12.1%)
PC2 (7.6%)
WT
KO
Day 3
Day 5
Day 7
E
Day3
Day5
Day7
WT1
WT2
WT3
cKO1
cKO2
cKO3
Hist1h3a
Hist1h3b
Hist1h3c
Hist1h3d
Hist1h3e
Hist1h3f
Hist1h3g
Hist1h3h
Hist1h3i
Hist2h3b
Hist2h3c2
row min
row max
F
G
H
Normalized TPM
I
BM cells(Day0)
(H3.3fl/fl and H3.3fl/fl:LysMcre/cre)
20% L929
conditioned medium
BMDM
(Day 2)
(Day 4)
(Day 6)
15 hrs BrdU
(50 μg/ml)
24 hrs_BrdU
Flow cytometry analysis
(Day3)
(Day5)
(Day7)

Figure S3. Surface marker expression, *H3.3* deletion, *H3.1/2* gene expression on day3, 5 ,7 and BrdU incorporation schematic in WT and cKO cells, Related to Figure 1

A. Representative flow cytometry plots for CD11b and F4/80 expression in WT and cKO cells on day3, 5 and day7.
B. RNA-seq normalized read counts of *H3f3a* gene in WT and cKO cells at different time points. n (number of biological replicates) =3
C. RNA-seq normalized read counts of *H3f3b* gene in WT and cKO cells at different time points. n (number of biological replicates) =3
   Note: H3f3b gene deletion was found to be fluctuating as we observed complete deletion as well as partial deletion of the gene in different cKO samples.
D. Principal component analysis (PCA) to evaluate global transcriptional differences between wild-type (WT) and knockout (KO) samples on day 3, 5, and 7.
E. TPM values of core histone *H3.1/2* genes in WT and cKO cells on day3, 5 and day7.
F. The bar graph illustrates the difference in TPM values for core histone H3.1/2 genes in WT versus cKO cells on day 3. n (number of biological replicates) =3, unpaired t test was used to calculate p values. (*: $p < 0.05$, ns – Not significant).
G. TPM values of core histone *H3.1/2* genes in WT and cKO cells on day5. n (number of biological replicates) =3 (unpaired t test, ns – Not significant)
H. Bar graph shows comparison of TPM values of core histone *H3.1/2* genes in WT and cKO cells on day7. N (number of biological replicates) =3 (unpaired t test, ns – Not significant)
I. Schematic representation of BrdU incorporation in WT and cKO cells.

Figure S4

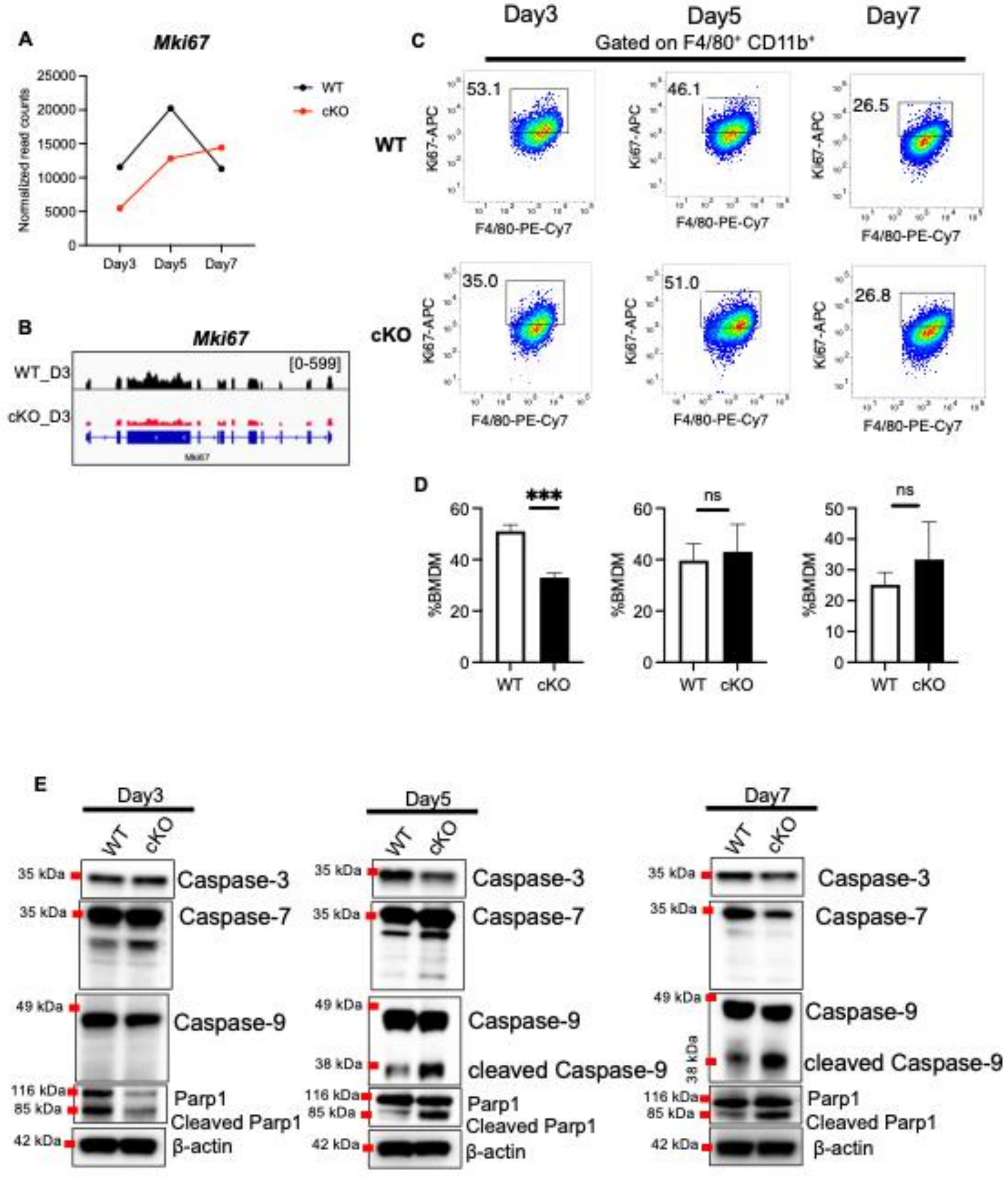
A
Mki67
Normalized read counts
25000
20000
15000
10000
5000
0
Day3
Day5
Day7
WT
cKO
B
Mki67
WT_D3
[0-599]
cKO_D3
C
Day3
Day5
Day7
Gated on F4/80+ CD11b+
WT
cKO
53.1
46.1
26.5
35.0
51.0
26.8
Ki67-APC
F4/80-PE-Cy7
D
%BMDM
***
ns
ns
WT
cKO
E
Day3
Day5
Day7
WT
cKO
35 kDa
Caspase-3
35 kDa
Caspase-7
49 kDa
Caspase-9
38 kDa
cleaved Caspase-9
116 kDa
Parp1
85 kDa
Cleaved Parp1
42 kDa
β-actin

Figure S4. Mki67 expression and apoptosis in cKO cells, Related to Figure 1

A. Graph shows difference in RNA seq average read counts (normalized) of *Mki67* between WT and cKO cells
B. RNA-seq IGV browser screenshot shows lower expression of *Mki67* RNA on day3 in cKO cells.
C. Representative plots for flow cytometry analysis of Ki67 protein expression in WT and cKO cells on day3,5 and 7.
D. % BMDM represent the average of three WT and three *H3.3* cKO mice ± SD, n (number of biological replicates) =3. p values were calculated using unpaired t test (***: $p < 0.001$; ns – Not significant).
E. Cleavage of Parp1 and caspase proteins are shown through immunoblotting of WT and cKO whole cell extract.

Figure S5

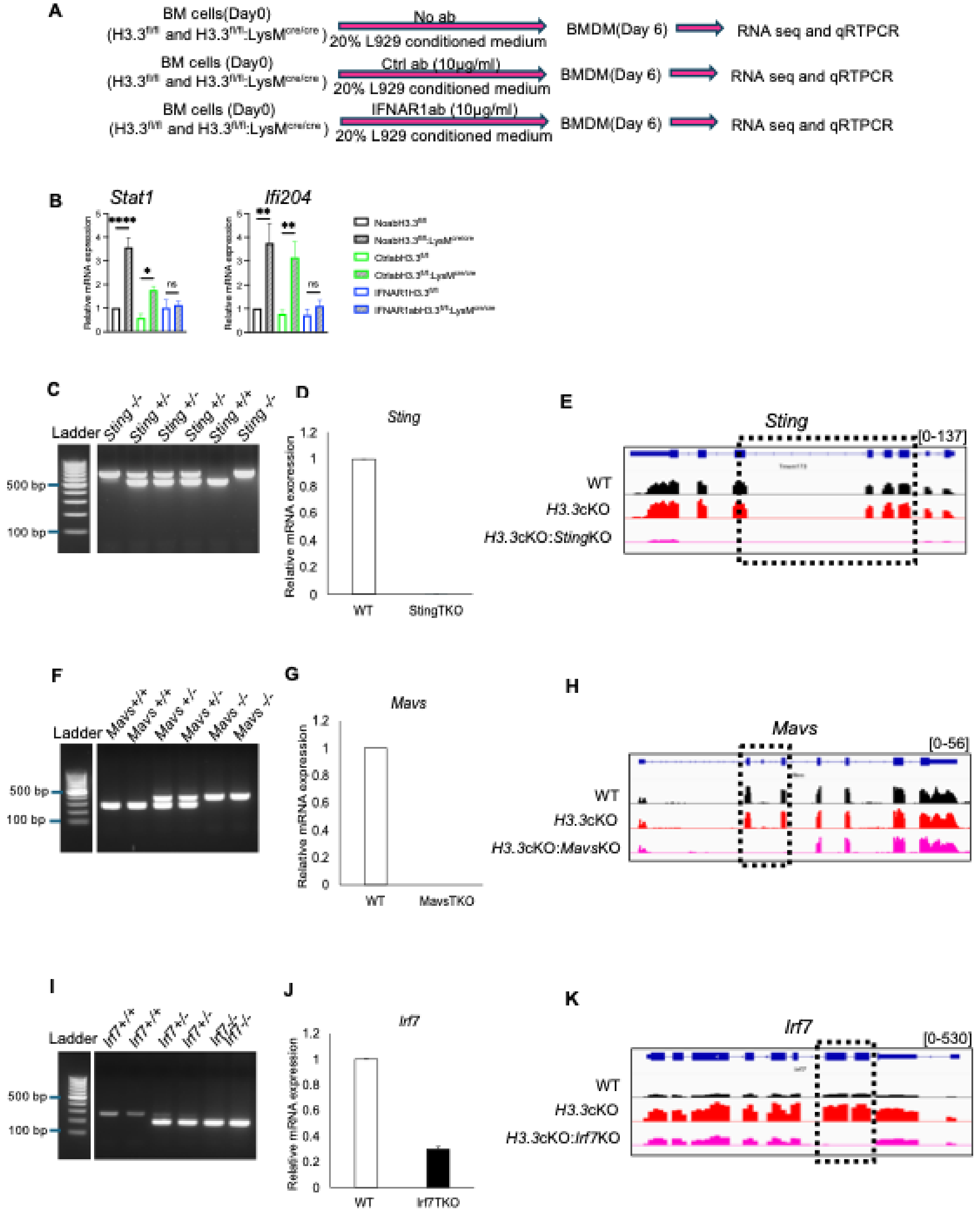
A
BM cells(Day0)
(H3.3fl/fl and H3.3fl/fl:LysMcre/cre)
No ab
20% L929 conditioned medium
BMDM(Day 6)
RNA seq and qRTPCR
BM cells (Day0)
(H3.3fl/fl and H3.3fl/fl:LysMcre/cre)
Ctrl ab (10µg/ml)
20% L929 conditioned medium
BMDM(Day 6)
RNA seq and qRTPCR
BM cells (Day0)
(H3.3fl/fl and H3.3fl/fl:LysMcre/cre)
IFNAR1ab (10µg/ml)
20% L929 conditioned medium
BMDM(Day 6)
RNA seq and qRTPCR
B
Stat1
Ifi204
Relative mRNA expression
ns
C
Ladder
Sting -/-
Sting +/-
Sting +/-
Sting +/-
Sting +/+
Sting -/-
500 bp
100 bp
D
Sting
Relative mRNA expression
WT
StingTKO
E
Sting
[0-137]
WT
H3.3cKO
H3.3cKO:StingKO
F
Ladder
Mavs+/+
Mavs +/+
Mavs +/-
Mavs +/-
Mavs -/-
Mavs -/-
500 bp
100 bp
G
Mavs
Relative mRNA expression
WT
MavsTKO
H
Mavs
[0-56]
WT
H3.3cKO
H3.3cKO:MavsKO
I
Ladder
Irf7+/+
Irf7+/+
Irf7+/-
Irf7+/-
Irf7-/-
Irf7-/-
500 bp
100 bp
J
Irf7
Relative mRNA expression
WT
Irf7TKO
K
Irf7
[0-530]
WT
H3.3cKO
H3.3cKO:Irf7KO

Figure S5. ISG upregulation in cKO cells is dependent on interferon feedback, Related to Figure 5

A. Schematic representation of IFNAR assay

B. qRT-PCR based confirmation of IFNAR assay by using *Ifi204* and *Stat1* genes. n (number of biological replicates) =3 mice. p values were calculated using ordinary one-way ANOVA test ((****: $p < 0.0001$; **: $p < 0.01$; *: $p < 0.05$, ns - Not significant). The error bars represent standard error of mean.

C. Confirmation of *Sting* gene deletion by genomic DNA PCR in *Sting* KO BMDMs.

D. qRT-PCR confirmed deletion of *Sting* gene in *Sting* TKO (*H3.3*$^{fl/fl}$:*LysM*$^{cre/cre}$:*StingKO)* BMDMs.

E. RNA-seq based IGV track confirmed *Sting* gene deletion in H*3.3*$^{fl/fl}$:*LysM*$^{cre/cre}$:*StingKO* BMDMs. Dotted lines represent deleted genomic regions.

F. Genotyping validation of *Mavs* gene deletion via genomic DNA PCR in *Mavs* KO BMDMs.

G. Quantitative Real-Time PCR with *Mavs* TKO (*H3.3*$^{fl/fl}$:*LysM*$^{cre/cre}$: *MavsKO)* BMDMs validated the deletion of *Mavs* gene.

H. RNA-seq gene tracks show deletion of *Mavs* gene exons in *H3.3*$^{fl/fl}$:*LysM*$^{cre/cre}$: *MavsKO* BMDMs. Dotted lines represent deleted genomic regions.

I. Genomic DNA PCR based verification of *Irf7* gene deletion in *Irf7* KO BMDMs.

J. qRT-PCR validation of *Irf7* gene deletion in *Irf7* TKO (*H3.3*$^{fl/fl}$:*LysM*$^{cre/cre}$: *Irf7KO)* BMDMs

K. RNA-seq gene tracks demonstrate deletion of the *Irf7* gene in *H3.3*$^{fl/fl}$:*LysM*$^{cre/cre}$: *Irf7KO* BMDMs. Dotted lines represent deleted genomic regions.

Figure S6

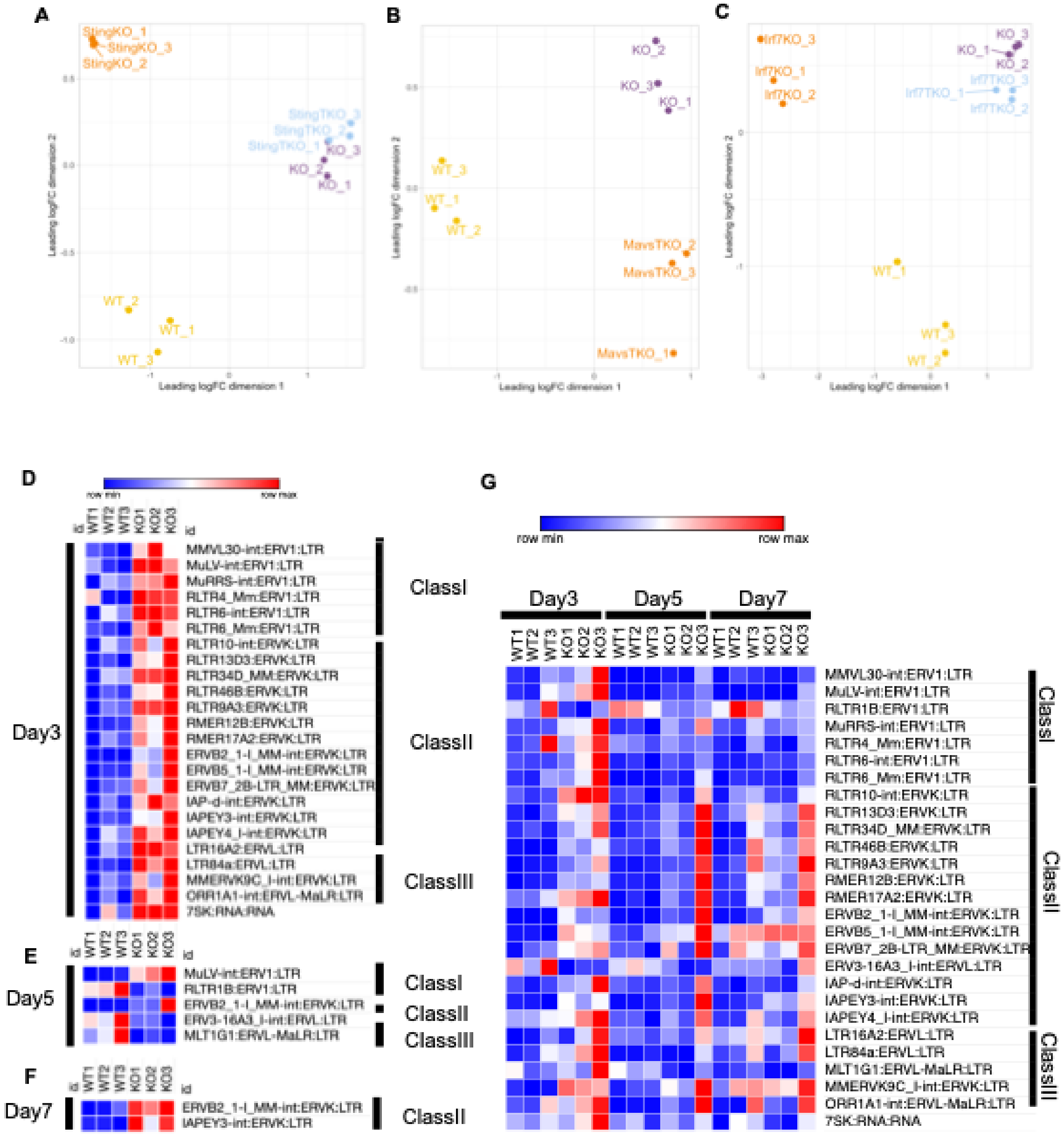
A
B
C
D
E
F
G
StingKO_1
StingKO_3
StingKO_2
StingTKO_3
StingTKO_2
StingTKO_1
KO_3
KO_2
KO_1
WT_2
WT_1
WT_3
MavsTKO_2
MavsTKO_3
MavsTKO_1
Irf7KO_3
Irf7KO_1
Irf7KO_2
Irf7TKO_3
Irf7TKO_1
Irf7TKO_2
Leading logFC dimension 1
Leading logFC dimension 2
row min
row max
Day3
Day5
Day7
ClassI
ClassII
ClassIII
WT1 WT2 WT3 KO1 KO2 KO3
MMVL30-int:ERV1:LTR
MuLV-int:ERV1:LTR
MuRRS-int:ERV1:LTR
RLTR4_Mm:ERV1:LTR
RLTR6-int:ERV1:LTR
RLTR6_Mm:ERV1:LTR
RLTR10-int:ERVK:LTR
RLTR13D3:ERVK:LTR
RLTR34D_MM:ERVK:LTR
RLTR46B:ERVK:LTR
RLTR9A3:ERVK:LTR
RMER12B:ERVK:LTR
RMER17A2:ERVK:LTR
ERVB2_1-I_MM-int:ERVK:LTR
ERVB5_1-I_MM-int:ERVK:LTR
ERVB7_2B-LTR_MM:ERVK:LTR
IAP-d-int:ERVK:LTR
IAPEY3-int:ERVK:LTR
IAPEY4_I-int:ERVK:LTR
LTR16A2:ERVL:LTR
LTR84a:ERVL:LTR
MMERVK9C_I-int:ERVK:LTR
ORR1A1-int:ERVL-MaLR:LTR
7SK:RNA:RNA
RLTR1B:ERV1:LTR
ERV3-16A3_I-int:ERVL:LTR
MLT1G1:ERVL-MaLR:LTR

Figure S6. MDS plots of WT, cKO, *Sting*/*Mavs*/*Irf7*KO:H3.3cKO RNA seq samples and ERV expression analysis in WT and cKO RNA seq samples, Related to Figure 5

A. MDS plot for WT, KO (*H3.3*cKO) and *Sting*TKO (*H3.3*cKO: *Sting*KO) RNA seq samples.
B. MDS plot for WT, KO (*H3.3*cKO) and *Mavs*TKO (H3.3cKO: *Mavs*KO) RNA seq samples.
C. MDS plot for WT, KO (*H3.3*cKO), *Irf7*KO and *Irf7*TKO (*H3.3*cKO: *Irf7*KO) RNA seq samples.
D. Heatmap demonstrates the RNA-seq normalized read counts of significantly altered ERVs in cKO vs. WT cells on day3.
E. RNA-seq normalized read counts for dysregulated ERVs in cKO vs. WT cells on day5.
F. RNA-seq normalized read counts of differentially expressed ERVs on day 7 in cKO vs. WT BMDMs.
G. Heatmap shows pattern of all the differentially expressed ERVs across day3, day5 and day7. We performed TEtranscripts and DEseq2 analysis with WT day3 RNA-seq sample as the control. This allowed normalization across all sample.

Figure S7

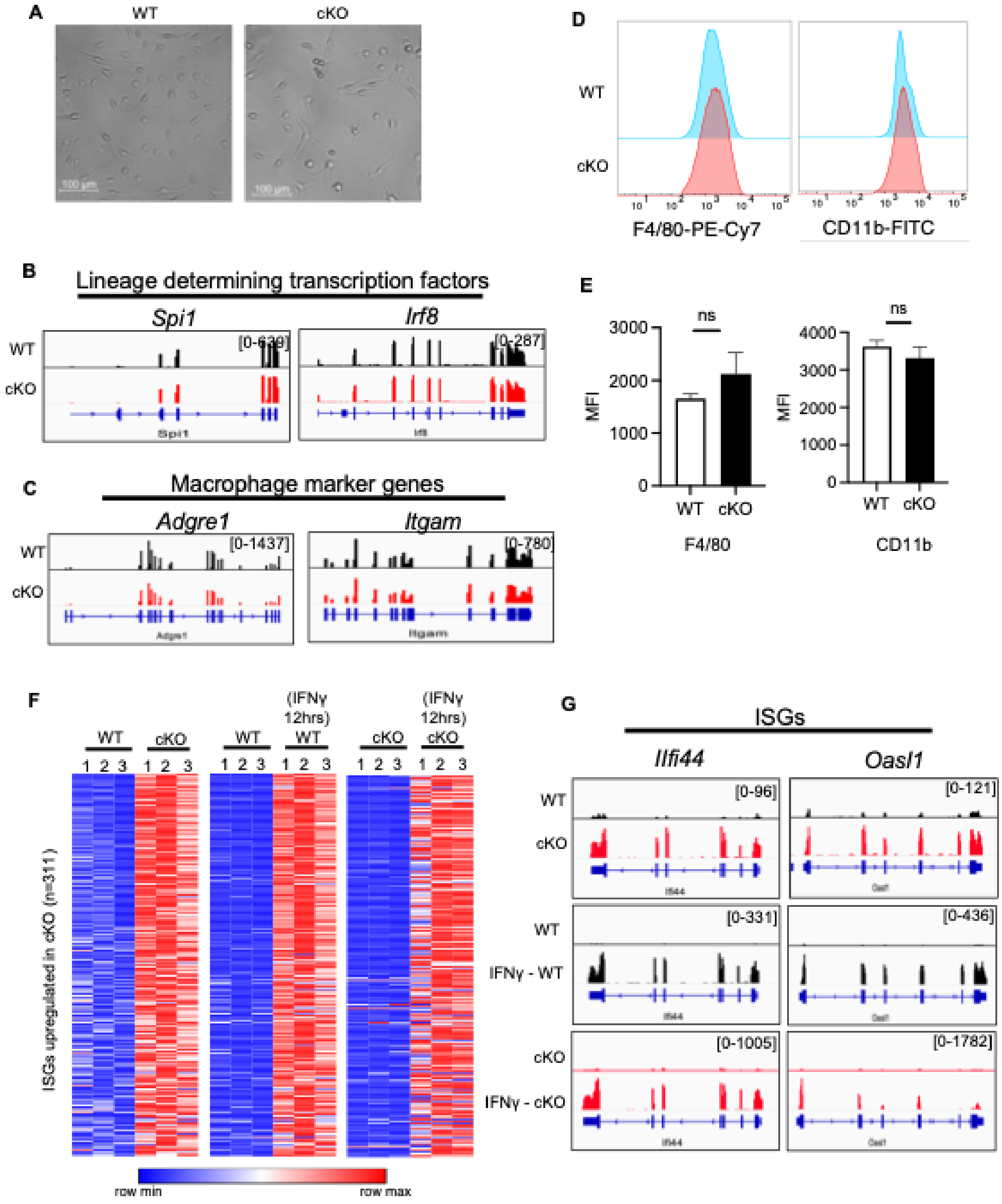
A
WT
cKO
100 µm
100 µm
D
WT
cKO
F4/80-PE-Cy7
CD11b-FITC
B
Lineage determining transcription factors
Spi1
Irf8
WT
cKO
[0-639]
[0-287]
E
ns
ns
MFI
MFI
3000
2000
1000
0
4000
3000
2000
1000
0
WT cKO
WT cKO
F4/80
CD11b
C
Macrophage marker genes
Adgre1
Itgam
WT
cKO
[0-1437]
[0-780]
F
WT
cKO
WT
(IFNγ 12hrs) WT
cKO
(IFNγ 12hrs) cKO
1 2 3
ISGs upregulated in cKO (n=311)
row min
row max
G
ISGs
Ilfi44
Oasl1
WT
cKO
[0-96]
[0-121]
WT
IFNγ - WT
[0-331]
[0-436]
cKO
IFNγ - cKO
[0-1005]
[0-1782]

Figure S7 *H3.3* deletion does not hinder M-CSF induced BMDM differentiation, Related to Figure1, Figure 3 and Figure 4

A. Brightfield microscopy images (scale bar =100μm) representing morphology of day7 live bone marrow derived macrophages from WT and cKO mice.
B. RNA-seq IGV gene tracks show equal expression of lineage determining transcription factors (*Spi1* and *Irf8*) in WT and cKO BMDMs.
C. Genic expression of macrophage marker gene *Adgre1/F4/80* and *Itgam/CD11b* in WT and cKO BMDMs.
D. Representative flow cytometry plots for staining of CD11b and F4/80 protein in WT and cKO cells.
E. MFI values were calculated for F4/80 and CD11b protein expression in WT and cKO cells, n (number of biological replicates) =3. Unpaired t test was used to calculate p values. (ns - Not significant)
F. Heatmaps depict upregulated normalized read counts of ISGs between WT vs cKO BMDMs, WT vs IFNγ treated (12hrs) WT BMDMs and cKO vs IFNγ treated (12hrs) cKO BMDMs.
G. RNA-seq IGV gene tracks show increased genic expression of *Ifi44* and *Oasl1* in WT vs cKO BMDMs, WT vs IFNγ treated (12hrs) WT BMDMs and cKO vs IFNγ treated (12hrs) cKO BMDMs.

Figure S8

A

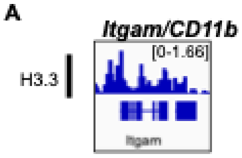

B

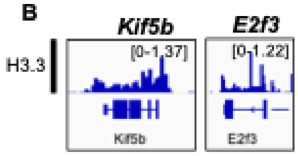

C

	GO terms	BinomP
Increased accessibility peaks	defense response to virus	5.86E-15
	positive regulation of apoptotic process	2.99E-08
	ribonucleoprotein complex biogenesis	4.02E-08
	cellular response to DNA damage stimulus	5.14E-07
	regulation of G1/S transition of mitotic cell cycle	4.85E-06
	pyrimidine ribonucleoside catabolic process	1.27E-05
Decreased accessibility peaks	regulation of cell adhesion	1.37E-30
	regulation of leukocyte activation	2.96E-27
	positive regulation of immune system process	7.32E-27
	regulation of cell activation	4.31E-26
	vesicle-mediated transport	4.60E-25
	response to external biotic stimulus	2.08E-23
	lipid biosynthetic process	6.60E-10

D

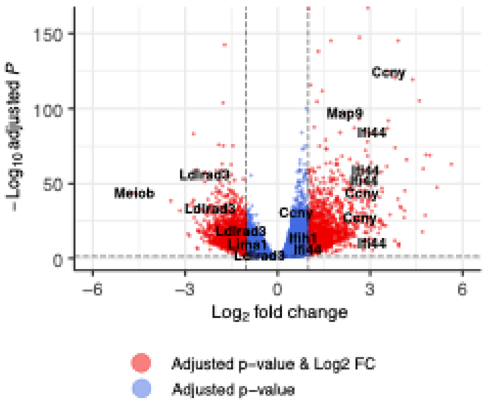

E

cKO gained motifs

Rank	Motif	P-value	T/B(%)	TF
1	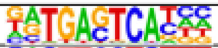	1e-286	36.02/8.28	Atf3(bZIP)(0.990)
2	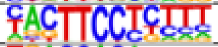	1e-161	37.02/13.82	Sfpi(0.953)
3	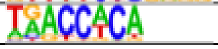	1e-61	31.42/16.94	Runx(0.914)

cKO lost motifs

Rank	Motif	P-value	T/B(%)	TF
1	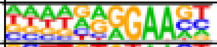	1e-623	67.11/20.58	Sfpi (0.906)
2	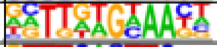	1e-81	37.71/21.86	CEBPD (0.887)
3	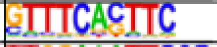	1e-74	8.58/2.08	Pu.1-IRF (0.943)
4	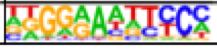	1e-73	13.79/4.89	NFkB-p65 (0.893)

Figure S8 H3.3 distribution and differential chromatin accessibility in WT and cKO cells, Related to Figure 6

A. CUT & RUN seq IGV gene track showing deposition of H3.3 on expressed gene *Itgam* in *H3f3b*-HA BMDMs.
B. CUT & RUN seq gene tracks showing deposition of H3.3 on cell cycle genes *Kif5b* and *E2f3* in *H3f3b*-HA BMDMs.
C. GO analysis of ATAC-seq increased and decreased accessible peaks in cKO BMDMs.
D. Volcano plot showing a correlation between ATAC seq peaks and RNA seq data. The nearest genes for increased or decreased ATAC peaks correlate with genes up or downregulated in *H3.3*cKO BMDMs.
E. Motif analysis of ATAC-seq KO gained, or KO lost peaks is shown.

Figure S9

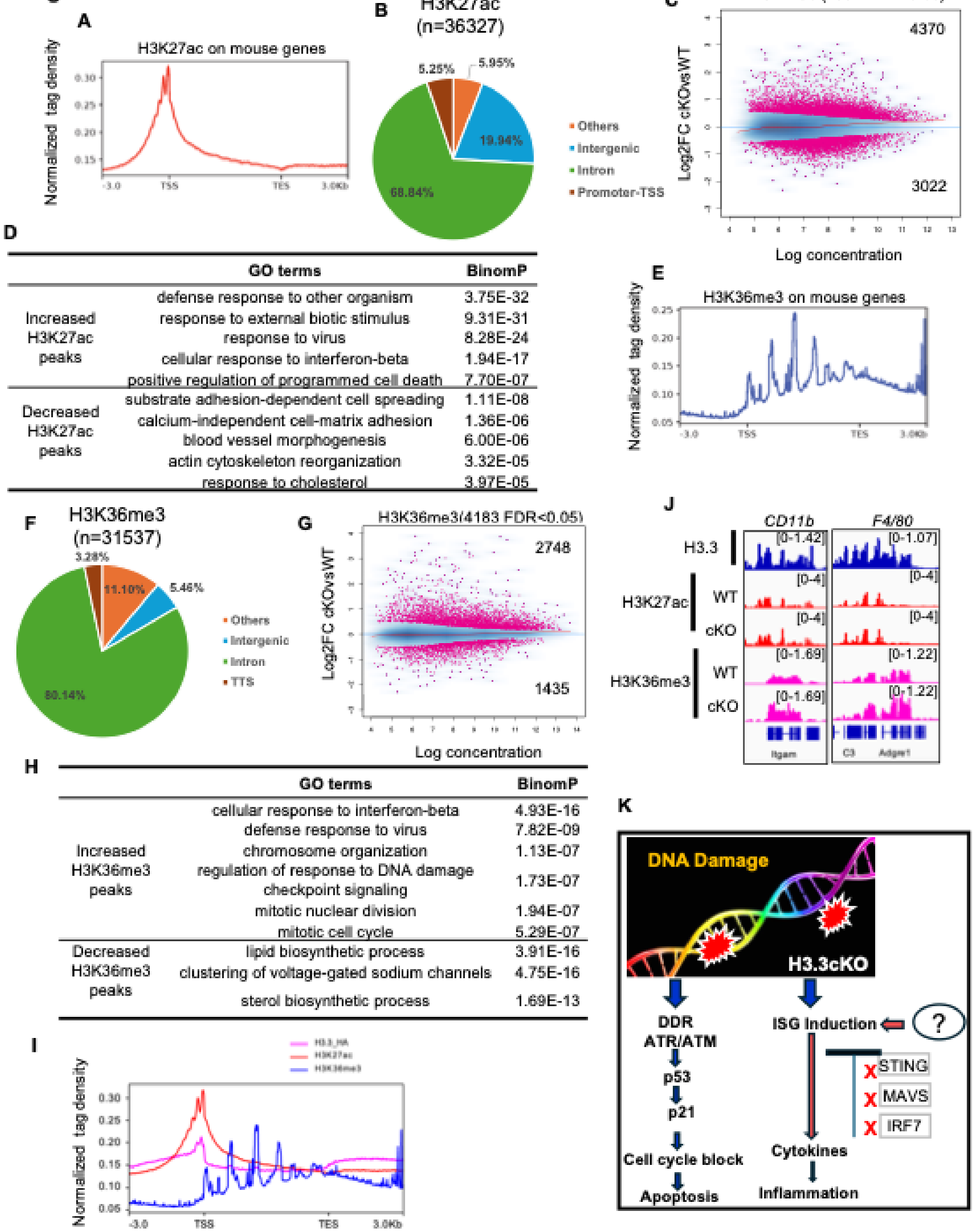

D

	GO terms	BinomP
Increased H3K27ac peaks	defense response to other organism	3.75E-32
	response to external biotic stimulus	9.31E-31
	response to virus	8.28E-24
	cellular response to interferon-beta	1.94E-17
	positive regulation of programmed cell death	7.70E-07
Decreased H3K27ac peaks	substrate adhesion-dependent cell spreading	1.11E-08
	calcium-independent cell-matrix adhesion	1.36E-06
	blood vessel morphogenesis	6.00E-06
	actin cytoskeleton reorganization	3.32E-05
	response to cholesterol	3.97E-05

H

	GO terms	BinomP
Increased H3K36me3 peaks	cellular response to interferon-beta	4.93E-16
	defense response to virus	7.82E-09
	chromosome organization	1.13E-07
	regulation of response to DNA damage checkpoint signaling	1.73E-07
	mitotic nuclear division	1.94E-07
	mitotic cell cycle	5.29E-07
Decreased H3K36me3 peaks	lipid biosynthetic process	3.91E-16
	clustering of voltage-gated sodium channels	4.75E-16
	sterol biosynthetic process	1.69E-13

Figure S9. Differential binding analysis of H3K27ac and H3K36me3, Related to Figure 6, 7

A. H3K27ac deposition on gene bodies +3kb/-3kb in WT BMDMs as per CUT & RUN seq in WT BMDMs.
B. Pie chart shows genome wide distribution of H3K27ac in WT BMDMs.
C. CUT & RUN seq MA plot represents increased or decreased H3K27ac peaks in cKO vs. WT BMDMs.
D. GO analysis of CUT & RUN seq KO gained and lost peaks of H3K27ac in cKO vs WT BMDMs.
E. CUT & RUN seq reveals H3K36me3 distribution on gene bodies in WT BMDMs.
F. Pie chart displays the genomic landscape of H3K36me3 in WT BMDMs.
G. CUT & RUN seq MA plot for H3K36me differential binding in cKO vs. WT BMDMs
H. GO analysis of KO gained and lost peaks of H3K36me3 in cKO vs WT BMDMs.
I. H3.3, H3K27ac and H3K36me3 distribution across known mouse genes in *H3f3b*-HA BMDMs (For H3.3) and WT BMDMs (*H3.3*$^{fl/fl}$ BMDMs - For H3K27ac and H3K36me3) as per CUT & RUN seq.
J. H3.3, H3K27ac and H3K36me3 distribution on macrophage marker genes *Itgam*, *Adgre1* in *H3f3b*-HA BMDMs (For H3.3) and WT BMDMs (*H3.3*$^{fl/fl}$ - For H3K27ac and H3K36me3) as obtained by CUT & RUN seq.
K. Activation of DDR and ISG induction upon DNA damage in *H3.3*cKO cells.

   Left: Canonical DDR activation in *H3.3*cKO cells - ATR, ATM and p53 are phosphorylated in *H3.3*cKO cells. p21, a downstream target of phospho-p53 is expressed in *H3.3*cKO cell, leading to cell cycle arrest and apoptosis. Consequently, much fewer macrophages were produced from *H3.3*cKO progenitors.

   Right: DNA damage activates expression of numerous ISGs, including inflammatory cytokines. This renders *H3.3*cKO macrophages and surrounding environment inflammatory. ISG induction in *H3.3*cKO cells is not mediated by STING, MARVS or IRF7. Another mechanism (a circle with a question mark) is responsible for ISG induction in *H3.3*cKO cells.

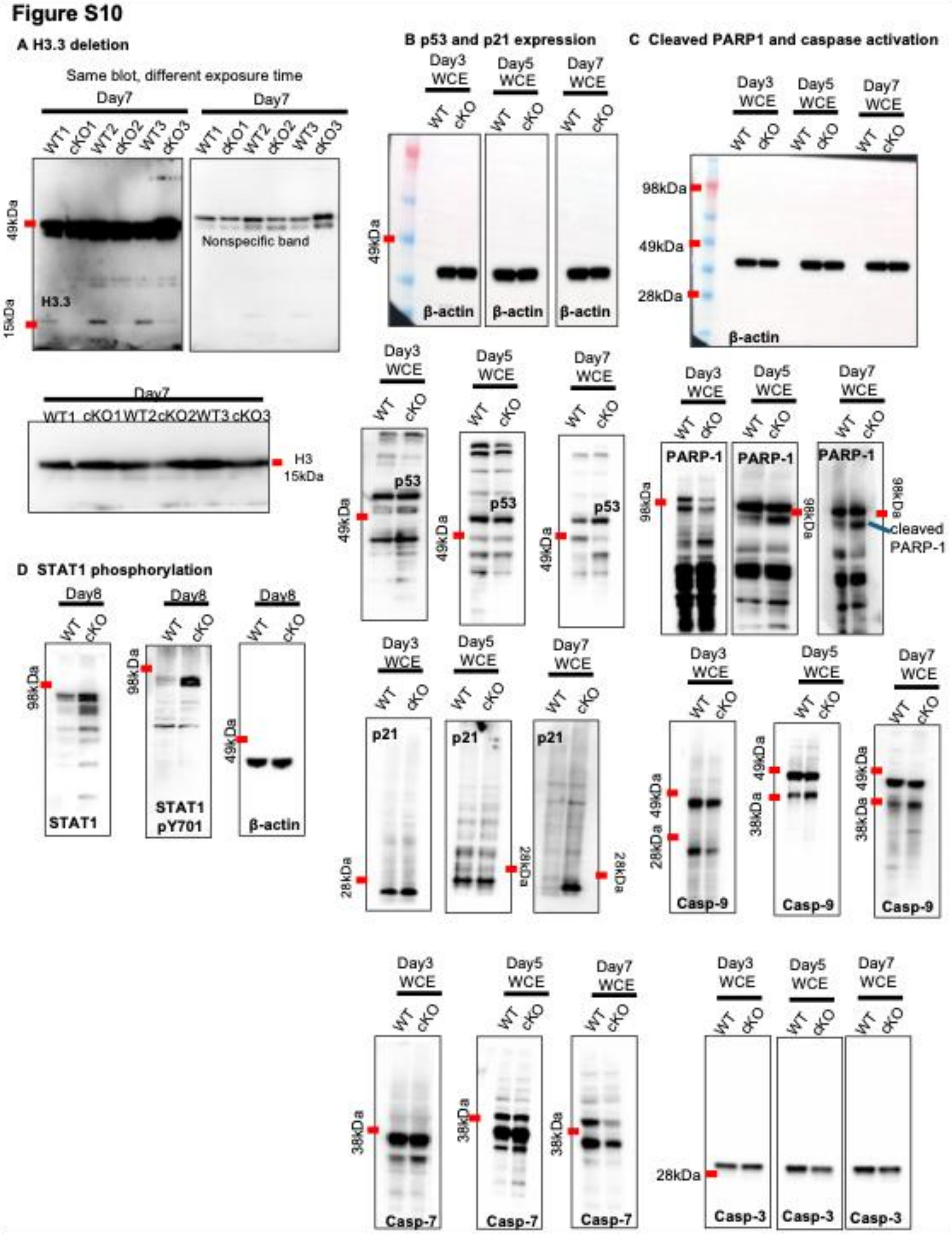
Figure S10
A H3.3 deletion
Same blot, different exposure time
Day7
WT1 cKO1 WT2 cKO2 WT3 cKO3
49kDa
15kDa
H3.3
Nonspecific band
H3
15kDa
B p53 and p21 expression
Day3 WCE
Day5 WCE
Day7 WCE
WT cKO
β-actin
p53
p21
28kDa
C Cleaved PARP1 and caspase activation
98kDa
28kDa
PARP-1
cleaved PARP-1
Casp-9
38kDa
Casp-7
Casp-3
D STAT1 phosphorylation
Day8
STAT1
STAT1 pY701

Figure S10 Raw data of western blots, Related to Figures S2, 2, 4, and S4.

A. Immunoblot to confirm H3.3 deletion in day 7 WT and cKO BMDMs as shown in Figure S2C.
B. Western blotting of p53, p21 with WT and cKO whole cell extract as shown in Figure 2E.
C. Western blotting of PARP1, Caspase9,7 and 3 with WT and cKO BMDMs (whole cell extract) as shown in Figure S4.
D. Immunoblot to show STAT1 overexpression and phosphorylation in WT and cKO BMDMs related to Figure 4.

Table S1. Cre and Flox excision PCR primers for *H3f3a* and *H3f3b* genes, Related to Figure 1

H3f3b	Cre P1 138160	TGGGATTAAAGAAATGTGTGTGCCAGC
	Cre P2 138161	GGATCCAAAATTCAAGGTCATTCACAGC
	Cre P3 138162	CTCCCCACCACTTGTGAATGTAAAACATTT
H3f3a	Cre P1 138090	CAAACAAGTTTTCAAATGTAGTTATGACTGATGC
	Cre P2 138091	CTAAACATACAAAACGTGGGAACACATTAGC
	Cre P3138099	AAGGGAATCGGATACGTTTCCAATTG
H3f3b	Flp 138154	AGGAGTGTTAATGCTGTTGCTCTTGTCCTC
	Flp 138155	TCACATCACTGAGGTCTGTGAACAGTCAGT
	Flp 138156	CCTCACCTTGTCGTATTATACTATGCCGATATACT
H3f3a	Flp 138094	CGACCTTTCTGTGTTTGTGGCTTCG
	Flp 138095	GATTTGCGGGCAGTCTGCTTTGTAC
	Flp 138097	CTGTGCTCGACGTTGTCACTGAAGC

Table S2. qRT-PCR primers, Related to Figure 1 and Figure 5

Gene Name (FP exon - RP exon)	FP	RP
H3f3a (Exon1-2)	CAGCGCCGCCTCTCGCTTG	CAGTCTGCTTTGTACGAGCCATGGTA
H3f3a (Exon2-3)	CTACAAAAGCCGCTCGCAAGAGT	TTCTGATAGCGTCTGATTTCACGG
H3f3b (Exon1-2)	CGATTGCGGCTCTTGTTCGAG	TTCCCACCGGTGGACTTCCTA
H3f3b (Exon2-3)	CCAAGGCGGCTCGGAAAAGC	GGTAACGACGGATCTCTCTCAGA
Irf7 (Exon5-7)	GGAGCTTGGATCTACTGTGGG	ATGCTGCATAGGGTTCCTCG
Sting (Exon4-5)	TATACCTCAGTTGGATGTTTGGC	CTGGAGTCAAGCTCTGAAGGC
Mavs (Exon3-4)	CTGGCTGATCAAGTGACTCG	AATGCAGAGGGTCCAGAAAC
Gtf2b	TACATGGCTTCCCAGGCTTC	CCCGATTTCTTTCTGTGTTCG

Table S7. Genotyping primers for *Sting*, *Mavs* and *Irf7* genes, Related to Figure 5

Sting	WTExon2FP	CCGTGCTGGCATCAAGAATC
	WTExon2RP	TGGGCTGGGCTAAACACTTC
	LaczFP	GGCGGTGATTTTGGCGATAC
	LaczRP	AATGCGGGTCGCTTCACTTA
Mavs	Common	AGCCAAGATTCTAGAAGCTGAGAA
	Wild type reverse	TAGCTGTGAGGCAGGACAGGTAAGG
	Mutant reverse	GTGGAATGTGTGCGAGGCCAGAGGC
Irf7	Neo16c	TCGTGCTTTACGGCCGCTCCCGATTC
	UZUcountFl	GTGGTACCCAGTCCTCTTTATAATCT
	R813long	AGTAGATCCAAGCGCTAAGTTCGTAC
